# Rating and ranking preparedness characteristics important for veterinary workplace clinical training: a novel application of pairwise comparisons and the Elo algorithm

**DOI:** 10.3389/fmed.2023.1128058

**Published:** 2023-04-21

**Authors:** Jennifer Routh, Sharmini Julita Paramasivam, Peter Cockcroft, Sarah Wood, John Remnant, Cornélie Westermann, Alison Reid, Patricia Pawson, Sheena Warman, Vishna Devi Nadarajah, Kamalan Jeevaratnam

**Affiliations:** ^1^School of Veterinary Medicine, University of Surrey, Guildford, United Kingdom; ^2^Bristol Veterinary School, University of Bristol, Langford, United Kingdom; ^3^School of Veterinary Medicine and Science, University of Nottingham, Sutton Bonington, United Kingdom; ^4^Faculty of Veterinary Medicine, Utrecht University, Utrecht, Netherlands; ^5^School of Veterinary Science, University of Liverpool, Neston, United Kingdom; ^6^School of Biodiversity, One Health and Veterinary Medicine, University of Glasgow, Glasgow, United Kingdom; ^7^Division of Human Biology, School of Medicine and IMU Centre for Education, International Medical University, Kuala Lumpur, Malaysia

**Keywords:** survey, questionnaire, Likert, comparison, rating, ranking, preparedness, methods

## Abstract

Quantitatively eliciting perspectives about a large number of similar entities (such as a list of competences) is a challenge for researchers in health professions education (HPE). Traditional survey methods may include using Likert items. However, a Likert item approach that generates *absolute* ratings of the entities may suffer from the “ceiling effect,” as ratings cluster at one end of the scale. This impacts on researchers’ ability to detect differences in ratings between the entities themselves and between respondent groups. This paper describes the use of pairwise comparison (this or that?) questions and a novel application of the Elo algorithm to generate *relative* ratings and rankings of a large number of entities, on a unidimensional scale. A study assessing the relative importance of 91 student “preparedness characteristics” for veterinary workplace clinical training (WCT) is presented as an example of this method in action. The Elo algorithm uses pairwise comparison responses to generate an importance rating for each preparedness characteristic on a scale from zero to one. This is continuous data with measurement variability which, by definition, spans an entire spectrum and is not susceptible to the ceiling effect. The output should allow for the detection of differences in perspectives between groups of survey respondents (such as students and workplace supervisors) which Likert ratings may be insensitive to. Additional advantages of the pairwise comparisons are their low susceptibility to systematic bias and measurement error, they can be quicker and arguably more engaging to complete than Likert items, and they should carry a low cognitive load for respondents. Methods for evaluating the validity and reliability of this survey design are also described. This paper presents a method that holds great potential for a diverse range of applications in HPE research. In the pursuit quantifying perspectives on survey items which are measured on a relative basis and a unidimensional scale (e.g., importance, priority, probability), this method is likely to be a valuable option.

## 1. Introduction

Surveys are popular in health professions education (HPE) research (1). In a bibliometric study of the three highest impact journals in HPE, 52% of research articles used at least one survey (2). Surveys are attractive because they can generate quantitative data about non-observable abstract ideas (constructs) such as respondents’ motivations, satisfaction, attitudes, preferences, values or beliefs (3).

When tasked with quantifying attitudes about a large number of entities in a single dimension, researchers in HPE may choose to ask research participants to rate each entity individually using closed questions with ordered response scales [Likert items (4, 5)]. However, if all the entities are judged to be fairly similar this presents a challenge for detecting differences in the ratings between the entities or between groups of participants. This paper presents an alternative methodology which generates relative ratings of entities across a unidimensional scale and facilitates between-group comparisons.

When symmetrical Likert items are used to measure attitudes such as student preparedness (6) or patient satisfaction (7), there is likely to be a skewed distribution of responses; a phenomenon called positivity bias (8). The ordinal and non-normally distributed data limits statistical analysis to non-parametric approaches (9). More importantly, when data clusters towards one of the limits (called the ceiling effect) this results in low measurement variability and reduces the researcher’s ability to detect differences in ratings between entities (10). Low rating spread between groups of respondents will limit the researchers’ ability to determine differences in perspectives between those groups and may lead to type II errors.

Further issues lie with the cognitive demands of answering a large number of Likert items, one per entity. Question context, particularly the questions immediately preceding the one at hand, affects the specific considerations that apply to each judgement, how respondents map their judgements onto the response scale, and how they edit their answers for consistency before reporting them (8). Contrast effects often seem to result when respondents are asked to judge many similar entities, successively, in a single dimension (8, 11, 12). This has been shown to be true when rating entities such as photographs for physical attractiveness (12) or human height (13), and when measuring subjective attitudes such as politician favourability (14). Thus, most attitude judgements are made on a relative basis (8, 15). This constant comparison process and response tracking, in addition to information retrieval concerning the entity itself, is likely to be cognitively demanding for respondents. The cognitive load will increase with the number of entities to be judged/compared (16) and respondents’ motivation to optimise responses will lose potency and exhaust them. This leads respondents to participate in a way that shortens response time: satisficing (17). Satisficing is a set of behaviours aiming to provide a satisfactory (rather than optimal) response, such as choosing the same answer for every question. Satisficing is likely when completing a large number of Likert items and it will introduce systematic bias and measurement error.

Therefore, there are several anticipated challenges for quantitatively measuring attitudes about a large number of entities on a unidimensional scale using Likert items, and in determining differences between groups of respondents (Table 1). There is a gap for an alternative which overcomes some of these issues.

**Table 1:** Advantages and disadvantages of using Likert items and pairwise comparisons to elicit perspectives about a large number of similar entities in a survey.

	Likert items	Pairwise comparisons
Advantages	Produces **absolute** ratings of the entities in question. The entities’ ratings can be labelled (e.g., “not important” or “very important”). The labels help to clarify the meaning of scale points (18). A method that’s familiar to HPE researchers and participants – few user instructions are required. Produce repeatable results provided inter-rater agreement is high and sample size is adequate. Questions can be combined to develop a Likert scale to explore a latent construct. Simple statistical approaches can be used to examine between-group differences.	Produces **relative** ratings of the entities in question. Produces continuous data; numerical differences in ratings are proportional to differences in attitudes. By definition, the ratings produced are spread along an entire scale. Measurement spread facilitates between-entity and between-group comparisons. In addition to the question order, the response option order can also be randomised. Therefore, they are not susceptible to systematic bias if respondents become fatigued. There is evidence that they are less complicated (19), more user-friendly (20), and quicker (19–21) for respondents to perform compared to Likert items in specific cases.
Disadvantages	Produces ordinal data and treating ordinal scales as interval scales is controversial (22). Susceptible to response clustering at the top of the rating continuum (positivity bias and the ceiling effect). High cognitive load associated with negotiating contrast effects and rating a large number of entities individually. Susceptible to satisficing, systematic bias and measurement error. Response option order cannot be randomised.	Making comparison judgements about entities which are very similar or not obviously comparable can be cognitively challenging for respondents. The ratings are not produced on a *per* participant basis so between-group comparisons require more complex statistical approaches. Beyond bipolar labels (a numerical rating of zero = “least” X and a rating of one = “most” X), the ratings produced cannot be attributed more detailed labels. Lack of familiarity for respondents, although they are intuitive.

Tourangeau et al. express that “if respondents find it difficult to map their judgements onto rating scales, then it may be possible to develop item formats that make the task easier, allowing them to answer more quickly, more reliably or both” (8) (p. 249). Pairwise comparisons are one such alternative. This question type involves presenting entities (or options) to respondents two at a time. The basic experimental unit is a comparison of the options and the respondent must choose one of them according to a specific criterion (e.g., the more important) (23). Pairwise comparisons are primarily used in cases where the entities cannot be directly measured, only subjectively judged. This is a common challenge in HPE research since it studies a fundamentally social phenomenon and stakeholder’s perspectives are frequently sought. Additionally, pairwise comparisons are an important heuristic that humans (subconsciously) perform on a daily basis, for example, the weather is better than yesterday, they are stronger than I am, this paper is more difficult than the last. In short, pairwise comparisons are how we measure things (24) and we seem to be psychologically wired for them (25).

Pairwise comparisons and Likert items have been compared in the wider literature with respect to specific use cases. Although ratings and rankings derived from pairwise comparison outcomes have been demonstrated to correlate strongly with Likert based results (19, 20, 26, 27), there are several advantages in using pairwise comparison questions to rate entities in a single dimension (Table 1). Pairwise comparisons are less complicated (19), user-friendly (20), and quicker (19–21) for respondents to perform compared to Likert items. It is easier for participants to assess two options at a time in comparisons, rather than handling all of the entities at once (28). The latter is an approach which is often employed when respondents alter their Likert item response selection for consistency throughout a survey (15). Considering response time as a proxy for cognitive load (29), there is tentative evidence that respondents find pairwise comparisons easier, and they prefer them (20, 21), possibly due to a gamification element. These aspects of pairwise comparisons, it is hypothesised, could improve participation, and reduce in-survey drop out.

In research settings when the true value of the attribute is known, such as biomedical image assessment, pairwise comparisons have been demonstrated to be even more accurate than Likert item scores (21). They have also demonstrated higher inter-rater reliability (20). Pairwise comparisons are efficient; a response to each question provides information about two different entities, whereas single Likert items only contribute information about one. Relatedly, there is also evidence that pairwise comparison-based methods require fewer participants to achieve equivalent results to rating based methods (19). Pairwise comparisons may, therefore, be well suited when a large number of entities are to be rated in an efficient manner.

It is inadequate to simply report the outcomes (the ‘winners’ and ‘losers’) of pairwise comparisons. The data must be processed to provide ratings and rankings of the entities. This requires a mathematical model. The ratings generated by such a model are a continuous data type rather than ordinal and the differences between ratings of the entities on the scale are proportional to the difference in the level of the attribute assigned to the entities by respondents. This is not necessarily applicable to Likert items (22). Likert items provide information about an entity on an absolute scale, whereas pairwise comparisons provide information on a relative scale. Therefore, if a research question is centred around comparing entities relatively, then pairwise comparisons are intuitively more suitable.

The Elo rating system (24) is a mathematical model derived by Arpad Elo to generate ratings and rankings of chess players using data from chess matches. It is not the only system that could be used to generate ratings and rankings of items from pairwise comparison data; there are a vast number of systems available (25) [e.g., Massey’s method (30), Colley’s method (31), Keener’s method (32), the Markov method (25), the Bradley-Terry model (33) or the Analytical Hierarchy Process (34)]. However, the underlying concept of the Elo rating system is simple, and it produces the output data required from the input data acquired: binary win/loss data. From online dating “apps” and primatology to urban planning and sports, the huge number of academic and industry applications (19, 35–41) not only demonstrate the Elo rating system’s flexibility but also relative ease of use in novel cases by non-mathematicians.

A novel application of the Elo rating system is proposed in this methodology paper. It is anticipated that the system can be readily modified to quantify attitudes about a large set of similar items in a single dimension. The items are treated like individual chess players, and each pairwise comparison that a respondent makes between two items can be seen as equivalent to a chess match. All items start with the same rating, but they diverge as successive pairwise comparisons take place. The Elo algorithm calculates an Elo rating for each item iteratively using the outcomes of every pairwise comparison made by respondents.

When sufficient pairwise comparison data has been entered into the algorithm, the Elo ratings will become relatively stable, and a ranking of the items can be produced. The ratings should form a stable ranking provided that (1) items are paired randomly, (2) there is sufficient variation in the items from the respondent’s perspectives and (3) there is a reasonable degree of shared perspectives among respondents in the group of study (19). A major advantage of the system is that, like in the global chess game, there is no requirement that every item is matched against every other. This is beneficial when there are a large number of items to rate (*n*), because the number of possible unique pair combinations quickly increases (*n* (*n* − 1)/2). In other words, it is possible for each survey respondent to perform pairwise comparisons on a subset of the items. Valuably, using the pairwise comparisons performed by participants of a particular demographic group (by pooling), Elo ratings and rankings can be produced on a group-by-group basis, which will generate continuous data for meaningful between-group comparisons.

### 1.1. Context in health professions education research: quantifying perspectives on preparedness for veterinary workplace clinical training case study

This paper presents a novel approach to assess perspectives on preparedness for veterinary workplace clinical training (WCT), utilising pairwise comparisons and the Elo algorithm. To the best of our knowledge this is the first instance where such a survey design has been employed in an HPE setting.

Insights from other health professions suggest that veterinary student preparedness for undergraduate WCT is important for several reasons; preparedness can impact students’ performance in the workplace (42–45), their stress surrounding the transition (46) and the job satisfaction of workplace supervisors (47). However, preparedness has not been characterised specifically for veterinary WCT, and which aspects are most important has not been evaluated. Since time and resources in HPE curricula are finite and investing into preparing students in one topic will come at the expense of another (48), schools need to be able to distinguish between the ‘very important’ and ‘important’.

Previous work (6) to explore pre-clinical veterinary students’ perspectives on preparedness for WCT used a pre-existing survey taken from the human HPE field (43, 49). Participants were asked to rate 62 preparedness characteristics in terms of their importance for veterinary WCT on a seven-point Likert item. Preparedness characteristics were assimilated into themes and all themes were rated as ‘important’ or ‘very important’. Significant differences in ratings could be identified at the theme level but the clustering of data around ‘important’ and ‘very important’ made distinguishing the relative importance on an individual characteristic level challenging. This limits the impact of the results and their usefulness in directing curriculum change to better prepare students for WCT.

Recent work by Judd et al. demonstrated that Likert items tended to render data indicating that *all* items concerning readiness for allied health clinical placements were important. They showed that students provided constrained ratings of the items compared to their educators, and suggest that students find it particularly difficult to differentiate between items or have a less nuanced understanding of preparedness (50). This provides further motivation for exploring an alternative survey design.

The overarching aim of this study is to determine the relative importance of aspects of preparedness for veterinary WCT. The objectives of this paper are (1) to describe the method of developing a pairwise comparison survey, (2) to describe use of the Elo rating system to generate ratings and rankings of the relative importance of preparedness characteristics for veterinary WCT and (3) to describe the method for gathering validity and reliability evidence about the survey.

## 2. Materials and equipment

An online platform was used to host the survey and gather the necessary data. The platform had several important features. Firstly, it had the facility to store a large bank of multiple-choice style questions (pairwise comparisons). Secondly, it was able to randomly allocate a subset of the pairwise comparisons to each participant. Thirdly, the subset could be administered to the participant in a random order, with the response options in a random order too. Finally, the platform could generate the following data: (1) a respondent identifier (an anonymous code), (2) the pairwise comparisons presented to each participant and (3) for each pairwise comparison completed, which response was selected by the participant (which item ‘won’) and which was not (‘lost’).

A computer with an integrated development environment (RStudio^1^) was used for data processing. The R package developed to produce the Elo ratings and rankings has been made publicly available.^2^ The EloChoice R package^3^ was required for the calculation of the weighted consistency index.

## 3. Methods

**Figure 1:**
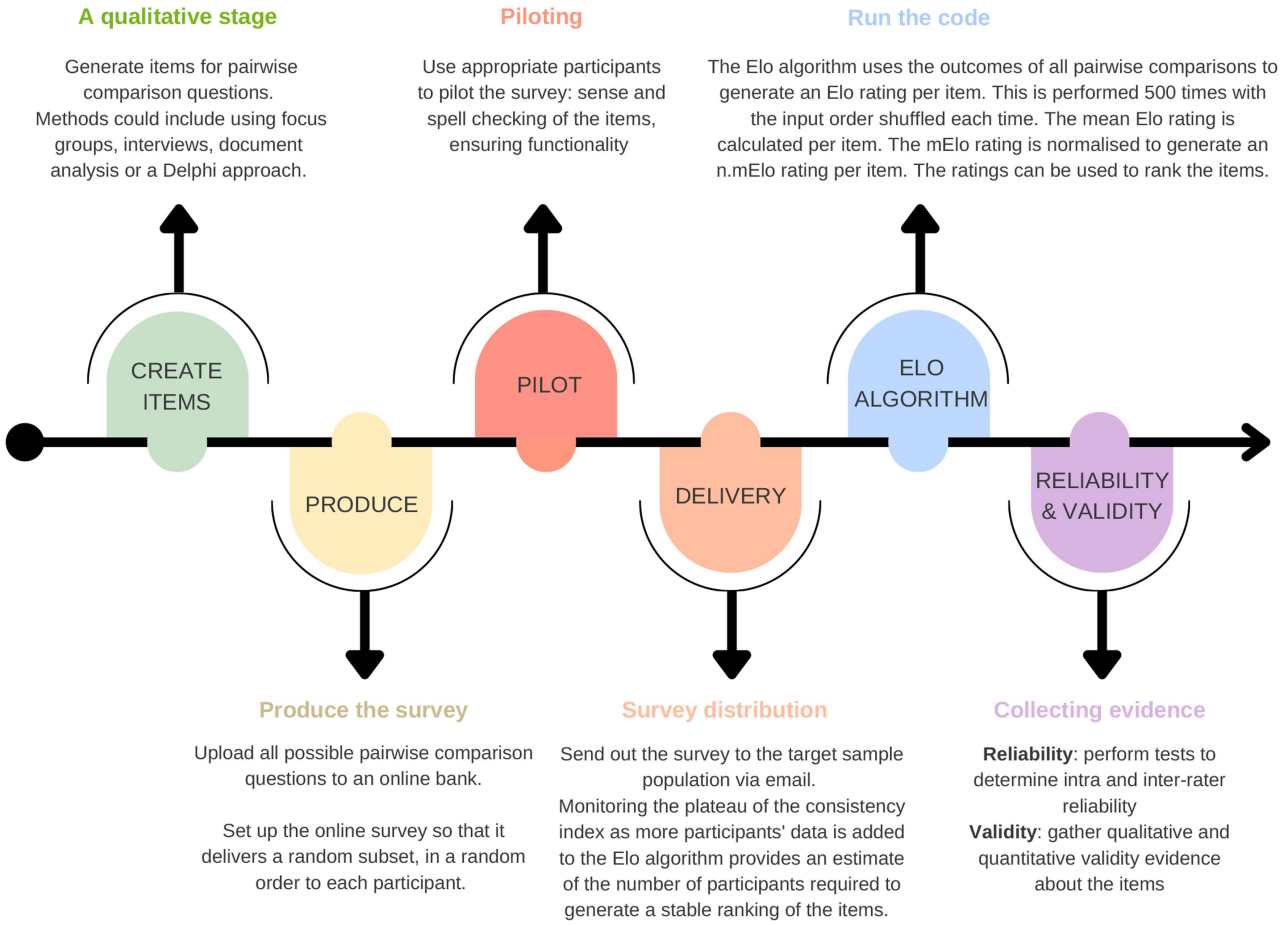
An overview of the methodology for producing a pairwise comparison survey and using the Elo algorithm to generate ratings and rankings of the items.

An overview of the method is provided in Figure 1.

### 3.1. Survey development and design

The pairwise comparison items (preparedness characteristics) were generated as part of a qualitative study aiming to characterise preparedness for veterinary WCT, and a detailed account of the methods and outcomes are published separately (51). Supplementary material 1 is a joint display (52) used to map the qualitative dimensions (participant quotes) to the survey’s items. It provides evidence for how the instrument was systematically developed. Further evaluation of the survey’s validity is described below.

There were 91 preparedness characteristics conceptualised as the knowledge, skills, personal attributes, awarenesses and behaviours which will facilitate student learning and working at the expected level during WCT. The 91 preparedness characteristics were organised into seven themes. The aim of the survey was to differentiate the relative importance of these preparedness characteristics.

**Figure 2:**
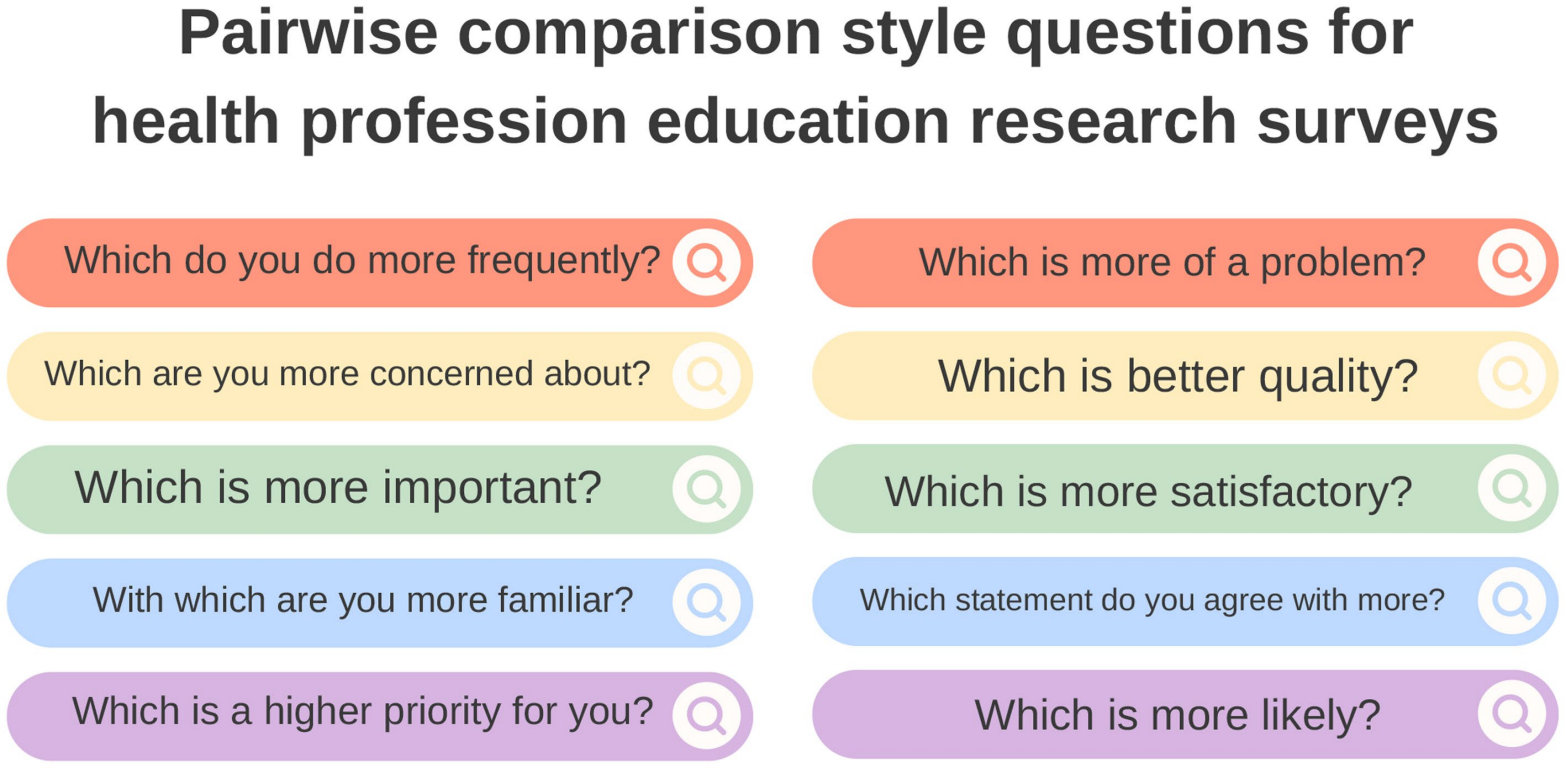
Example pairwise comparison style questions which could be utilised in health professions education (HPE) survey research.

The survey was hosted on Qualtrics.^4^ The question stem was the same every time: “Which characteristic do you think is *more* important for a veterinary student to be prepared for their workplace clinical training?” and two preparedness characteristics were presented for the participant to choose from, plus the option to select “I do not understand one or both of the options.” Alternative pairwise comparison style questions which could be used in HPE research are proposed in Figure 2. Each participant was randomly assigned fifty random pairwise comparisons to complete from a bank of all possible pair combinations (*n* = 4,095).

Additional components of the survey included an eligibility check against inclusion criteria, consent form, demographic information collection and pairwise comparison instructions.

Questionnaire piloting is discussed in the validity section.

### 3.2. Data collection

The survey was administered to clinical veterinary students that were due to commence WCT next at their school and workplace clinical supervisors. Sixty-five veterinary schools from a number of different countries were targeted for recruitment to take part in the survey. Twenty-six veterinary schools (40%) responded to requests to take part via email or virtual meetings. Local champions (senior academic members of faculty with responsibilities for research, clinical teaching and/or workplace learning) were sent the inclusion criteria and recruitment material to forward to the target cohort of students at their school via email or at events. Email reminders and response updates were sent periodically to local champions.

### 3.3. Data processing

Using the outcomes of the pooled pairwise comparisons completed by respondents in the survey, the Elo algorithm calculated an Elo rating for each preparedness characteristic. Instances where a respondent selected ‘I do not understand one or both of the options” were filtered out before commencing data processing.

All characteristics started with the same arbitrary rating (zero). In a sequential manner, the pooled pairwise comparisons were processed one by one by the algorithm, and the ratings of the two characteristics in the comparison increased or decreased depending on the outcome and the probabilities of each characteristic ‘winning’. An in-depth explanation of the mathematics underlying the system are provided in Supplementary material 2 (section 2). When sufficient pairwise comparisons are entered into the system, the characteristics’ ratings become relatively stable (19), and a ranking of the characteristics can be derived.

It was important to consider the order in which the pairwise comparisons were entered into the Elo algorithm for processing. With each entry of a pairwise comparison into the system, the subsequent update of the competing characteristics’ ratings was partially determined by the characteristics’ existing ratings (see Supplementary material 2 for the mathematical detail). Therefore, to achieve a more robust and less variable set of rankings (19), the mean Elo rating (mElo) for each characteristic was generated. This was calculated after the rating system was run with five hundred randomised, virtual sequences of the pooled pairwise comparisons.

Min-max normalisation (53) is a linear mathematical transformation which was performed on the original mElo ratings to generate ratings that ranged from zero to one (n.mElo ratings). An n.mElo rating of zero was attributed to the least important characteristic and an n.mElo rating of one was attributed to the most important characteristic. The 89 intermediary characteristics were attributed n.mElo ratings in-between this maximum and minimum, and the distances between the ratings were proportional to the original mElo rating differences. This normalisation process facilitates the comparison of sets of ratings generated from the perspectives of different groups of participants.

### 3.4. Survey validity

Validity is “the degree to which an instrument is measuring the construct it purports to measure” (54) (p. 743). Content validity is the degree to which the instrument has an appropriate set of items that reflect the full content of the target construct to be measured (55). This survey is intended to measure perspectives on the importance of preparedness characteristics for veterinary WCT. Given that the preparedness characteristics were generated from group interviews with stakeholders discussing preparedness for veterinary WCT, they are somewhat inherently valid for the survey. Nevertheless, mixed methods validation to review items’ content validity (55, 56) was performed.

Qualitative approaches were used to promote and assess validity. After the joint display was constructed from the group interview outcomes, the researchers involved in the survey development (JRo, KJ, SP, PC) reflected on the items individually, they performed 320 sample pairwise comparisons between them and debriefed as a team, discussing the relationship between the items (preparedness characteristics) and the construct to be measured (the relative importance of aspects of preparedness). The wording of some items was modified to improve their clarity. At this stage, no items were added or removed, nor their underlying content altered, because it was agreed that such decisions made by the research team would undermine the perspectives of the group interview participants.

The survey was piloted with University of Surrey academic staff (*n* = 7) and veterinary surgeons in practice who were familiar with supervising students in the workplace (*n* = 4). Feedback from the pilot was used to clarify some survey items further and correct spelling mistakes. There were no significant issues with functionality of the survey identified. Feedback from the pilot study led to the production and provision of a preparedness characteristic dictionary which was embedded in the survey and provided a more detailed descriptor of the item if required by participants.^5^

A quantitative approach to assessing validity was also employed after survey data collection ended. Content validity indices (55) (CVIs) were generated for all preparedness characteristics. Six of the research team who were not involved in the initial survey development but who are experienced clinical veterinary educationalists (JRe, CW, SWo, AR, PP, SWa) were asked to rate how relevant each characteristic was for the survey (not, somewhat, quite, or very relevant). The CVI was calculated as the proportion of raters who designated the characteristic as “quite” or “very relevant.” When an item’s average CVI is greater than 0.78 for three or more experts, this is good evidence of content validity (57).

### 3.5. Survey reliability

Reliability is consistency in measurement. Reliability evidence was gathered from (1) a reliability test performed by the same six veterinary educationalists (JRe, CW, SWo, AR, PP, SWa) and (2) from the main survey completed by veterinary students and clinical supervisors.

The reliability test used 39 randomly selected preparedness characteristics (out of 91). These were arranged into 13 triplets which were used to generate 52 pairwise comparison questions presented in a randomised order (for each triplet: preparedness characteristic A versus B, B versus C, A versus C, and B versus A [response option symmetry]). This test was repeated after 8 weeks.

Three measures of *intra*-rater consistency were calculated. Firstly, the proportion of instances when the same response was given to the pairwise comparison of A versus B and B versus A (response option symmetry). Secondly, the proportion of characteristic triplets which had transitive responses, e.g., where A was selected over B, B over C and A over C. Thirdly, the survey’s test–retest stability was measured using the proportion of instances when the same response was given to any pairwise comparison in the first and second (repeated) test.

*Inter*-rater consistency in pairwise comparison responses was calculated as the mean weighted consensus rate. This was calculated for the data in both the reliability test and the main survey. For every question that was responded to by more than one rater, a consensus rate was calculated as the proportion of instances where the mode response was given. For example, if a question was asked three times (to three different participants) and twice B was selected, the consensus rate for that question was 0.67. The mean weighted consensus rate was the average consensus rate across all of the questions, weighted for the number of times the question was responded to.

The significance of the reliability metrics was assessed against simulated data. Five thousand random responses to the reliability test were simulated and the inter/intra reliability metrics were calculated for those responses. The mean or median reliability metrics calculated from the simulated, random responses were compared to the test metrics using one sample t-tests or Wilcoxon one sample median tests, respectively, depending on the structure of the simulated data.

The data from the standalone reliability test were supported by a published consistency index (19) for pairwise comparison data processed using the Elo algorithm. The consistency index tracks how often pairwise comparison outcomes violate the expected outcome based on previous comparisons and according to the standing Elo ratings of the characteristics in question. The index is a value between zero and one, and one signifies perfect agreement between the pairwise comparison outcomes. A weighting can be applied to the index related to the degree to which any unexpected pairwise comparison outcomes are a surprise. Larger upsets, where the difference in existing Elo ratings between items is greater, will negatively impact the index more. The underlying mathematics of the index are explained in Supplementary material 2, section 4.3. The degree to which the index reflects inter- or intra-rater reliability is dependent on the ratio of raters to the number of times each item is compared by each rater.

To evaluate the reproducibility of the method, replicating the study and collecting new data to calculate a second set of n.mElo ratings of the preparedness characteristics would be the model approach. However, this undertaking exceeded the scope of what was feasible in the underlying population. Instead, a single demographic group’s data was randomly separated into two halves (*n* = 67 each). In essence, each half was treated as though the survey had been applied to two samples from the same underlying population. The Elo algorithm was used to generate a separate set of n.mElo ratings and rankings of the preparedness characteristics for each half. Subsequently, after checking the ratings for normality, Pearson’s and Kendall’s Tau correlation coefficients were calculated for the two sets of ratings and rankings, respectively. If the correlation was strong this was an indicator that the method produced reproducible results.

### 3.6. Sample size estimation

The accuracy of the weighted consistency index is expected to increase with the number of raters (19). As more raters perform more pairwise comparisons, this generates more input data for the algorithm and provides more information about the perspectives of the group. The weighted consistency index is also expected to plateau; beyond a point adding data from more raters will not improve the consistency index, or stabilise the preparedness characteristics’ rankings according to n.mElo ratings. Tracking the consistency index with increasing rater numbers can therefore be used to estimate the number of raters required to produce relatively stable rankings of the preparedness characteristics (19). This is achieved by calculating the weighted consistency index using only a single raters’ pairwise comparison data, then again with an additional rater’s data, and so on until all of the raters are included. Given that the order of rater inclusion could impact the outcomes, this process is repeated ten times with the order of rater inclusion randomised each time. The median weighted consistency index across the ten repeated trials, for each sample size, can be plotted and examined for the point of plateau. The plateau indicates that the consistency indices and rankings of n.mElo ratings are stable and provides evidence for the sample size required.

In order to allow for between-group comparisons of perspectives on preparedness for WCT in future work, recruitment efforts were based on attaining a sufficient number of responses *per* group of interest. Observations based on the consistency indices in a single demographic group (see Supplementary material 2, section 4.3) suggest that an absolute minimum of twenty participants, each performing fifty comparisons, are required to generate a stable ranking of the preparedness characteristics’ n.mElo ratings. In order to account for the possibility that raters in other demographic groups could have more diverse perspectives, lower inter-rater consistency and therefore a higher data requirement for the algorithm to generate stable set of rankings, forty participants per group was aimed for.

### 3.7. Ethical considerations

This study was granted ethical approval from the University of Surrey Ethics Committee (FHMS 20-21, 118 EGA Amend 2) 04/03/22. All participants were provided with a digital participant information sheet and gave written consent to take part in the study in the first section of the online survey. In instances where participants did not provide their consent the survey was automatically terminated. All participants were provided with an automated unique code upon completion. Within 7 days, if a participant chose to withdraw their data, they were able to email their code to the research team who could identify and remove their anonymous data from the data set. All participants were offered the opportunity to leave their contact details to take part in a prize draw for online shopping vouchers on completion.

## 4. Results

### 4.1. Metadata

**Figure 3:**
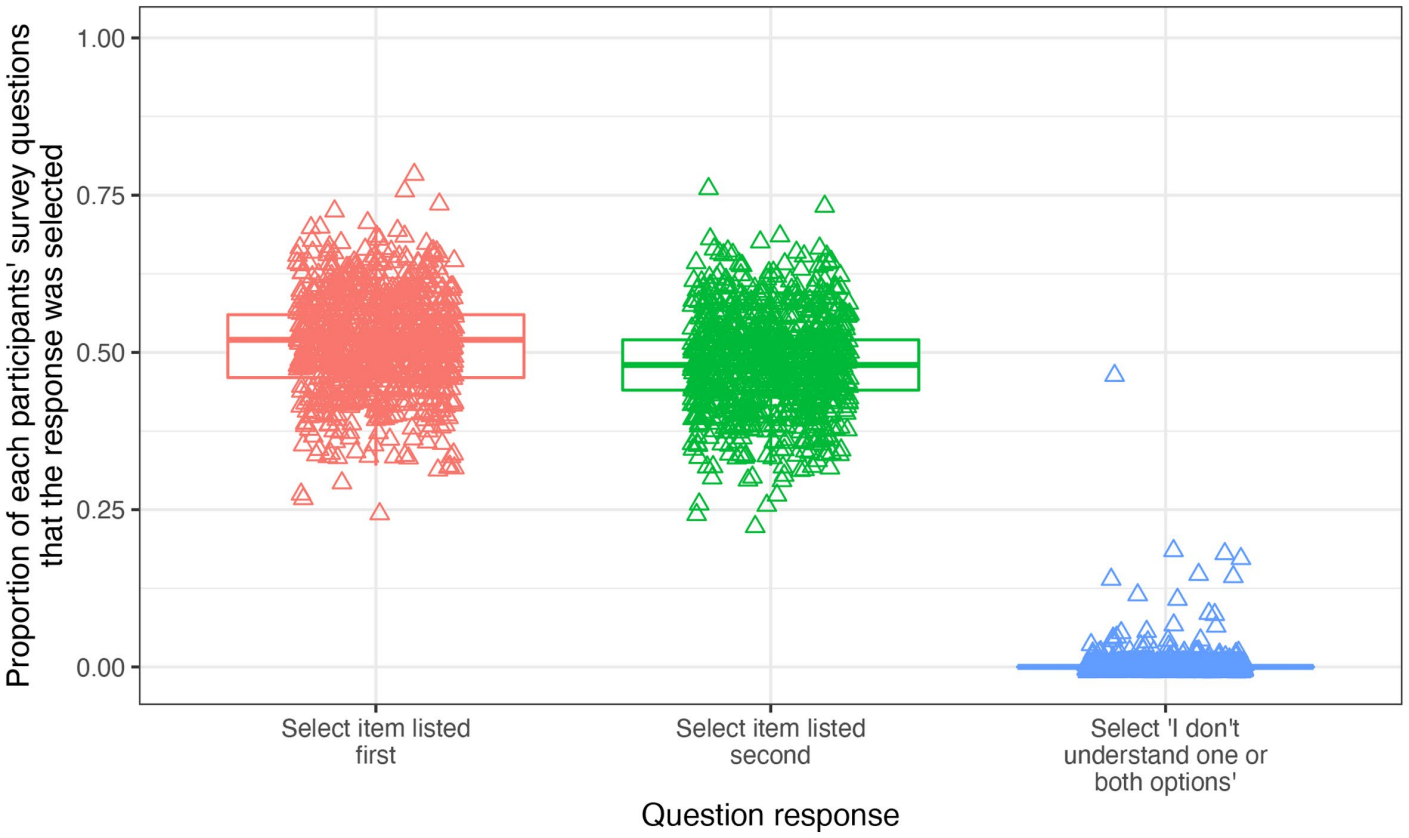
A chart to demonstrate the proportion of questions each response was selected by each survey participant. There are three box plots, one for each possible response in a pairwise comparison: selecting the item listed first, selecting item listed second or selecting “I do not understand one or both of the options”. Each participant is represented by a triangle on each box plot, indicating the proportion of times they selected each response across the survey.

Performing a total of 45,050 pairwise comparisons between them, 901 respondents completed the survey. In 51.6% of pairwise comparisons the first item presented was selected as the more important and the second item was selected in 48.1% of pairwise comparisons. The “I do not understand one or both of the options” response was selected in 0.34% of instances. The proportion of pairwise comparisons in which each response was selected, on an individual participant basis, is demonstrated in Figure 3.

**Figure 4:**
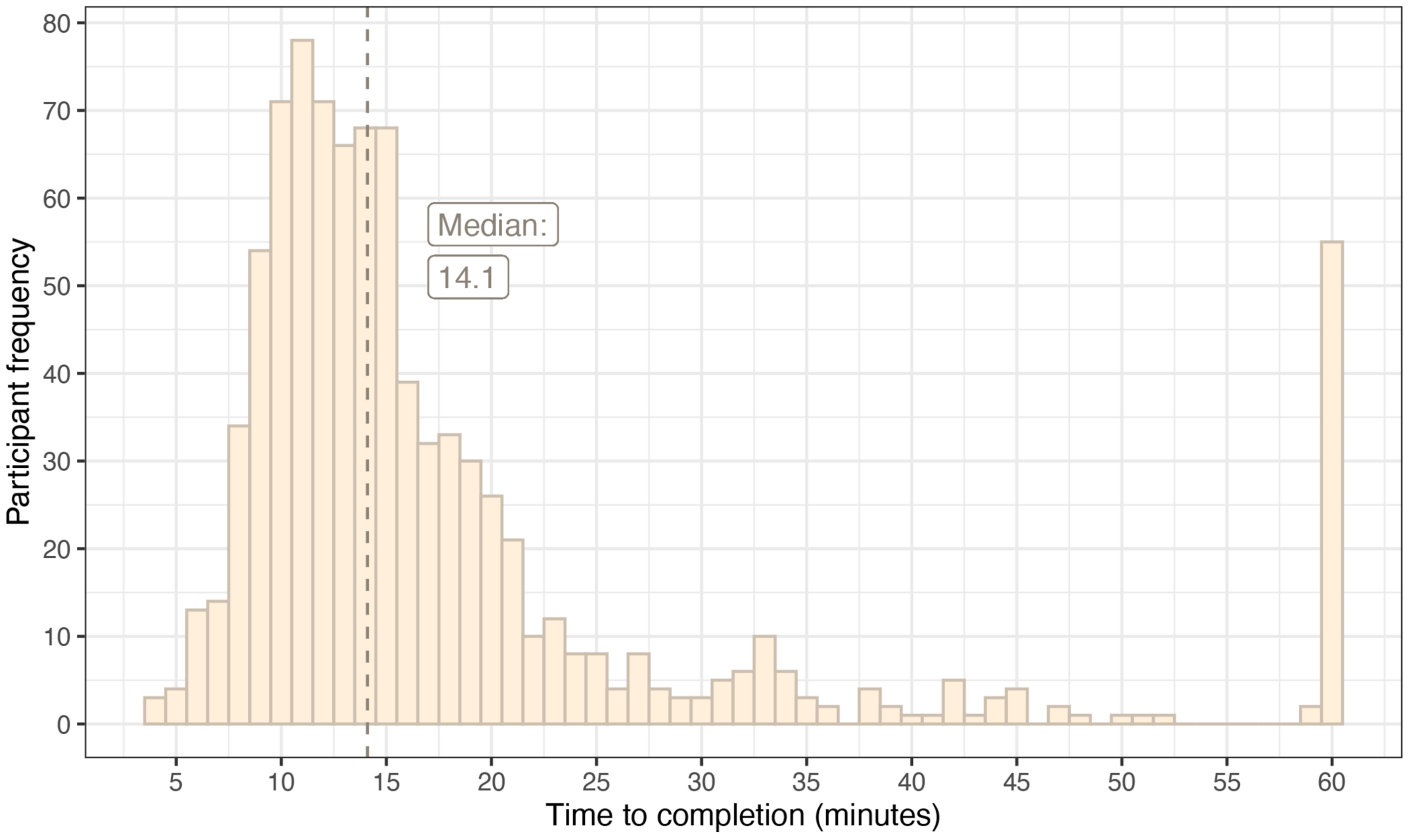
A histogram demonstrating the distribution of response times for the survey.

The median time to complete the survey was 14.1 minutes. Fifty-five participants took more than 1 hour, and it was assumed that these participants left the survey window open and returned to it at another time. These outliers were included in the median but were winsorized for the production of Figure 4 which demonstrates the distribution of response time.

### 4.2. n.mElo ratings and rankings of items (preparedness characteristics)

After generating five hundred virtual sequences of the completed pairwise comparisons, the Elo algorithm produced five hundred Elo ratings per item (per preparedness characteristic), which were averaged to generate a single mElo rating per item. The mElo ratings were min-max normalised to produce a n.mElo rating for each item. These values represent the relative importance of the item from the perspectives of the students and supervisors combined, with 1.000 being the most important characteristic and 0.000 being the least.

### 4.3. Median n.mElo ratings for the survey themes

The preparedness characteristics map to seven themes of preparedness (51). The median n.mElo rating for each theme is provided in Table 2 to demonstrate how ratings can be used to summarise data.

**Table 2:** Median n.mElo ratings and rankings of preparedness characteristics in seven themes of preparedness for veterinary workplace clinical training, according to the perspectives of all respondents.

Theme	Median n.mElo rating	Rank
Prepared with a growth mindset	0.670	1
Prepared for communication, consultation and clinical reasoning	0.661	2
Prepared for self-directed and experiential learning whilst working	0.645	3
Prepared with intrinsic motivation and enthusiasm for learning and working	0.553	4
Prepared with the practical competence and confidence for work	0.486	5
Prepared with the knowledge for work	0.381	6
Prepared for the transition to learning and working in a clinical and professional environment	0.356	7

### 4.4. Survey validity

The content validity indices (CVIs) across the items took values of 0.5 (*n* = 4), 0.66 (*n* = 13), 0.83 (*n* = 26) to 1.0 (*n* = 48), therefore 74 of the items (81%) had good evidence of content validity.

### 4.5. Survey reliability

#### 4.5.1. Intra-rater reliability

In the reliability test, the symmetry consistency was 0.92 (the proportion of instances where the same response was provided to a random pairwise comparison of preparedness characteristic A compared to B and then a second question where the item order was reversed: B compared to A). The proportion of pairwise comparison triplets which demonstrated transitivity in the responses was 0.97. In the second application of the reliability test 8 weeks after the first, the proportion of pairwise comparisons where the same response was provided by test respondents was 0.74. There was individual variation between respondents, ranging from 0.65 to 0.85.

#### 4.5.2. Inter-rater reliability

**Figure 5:**
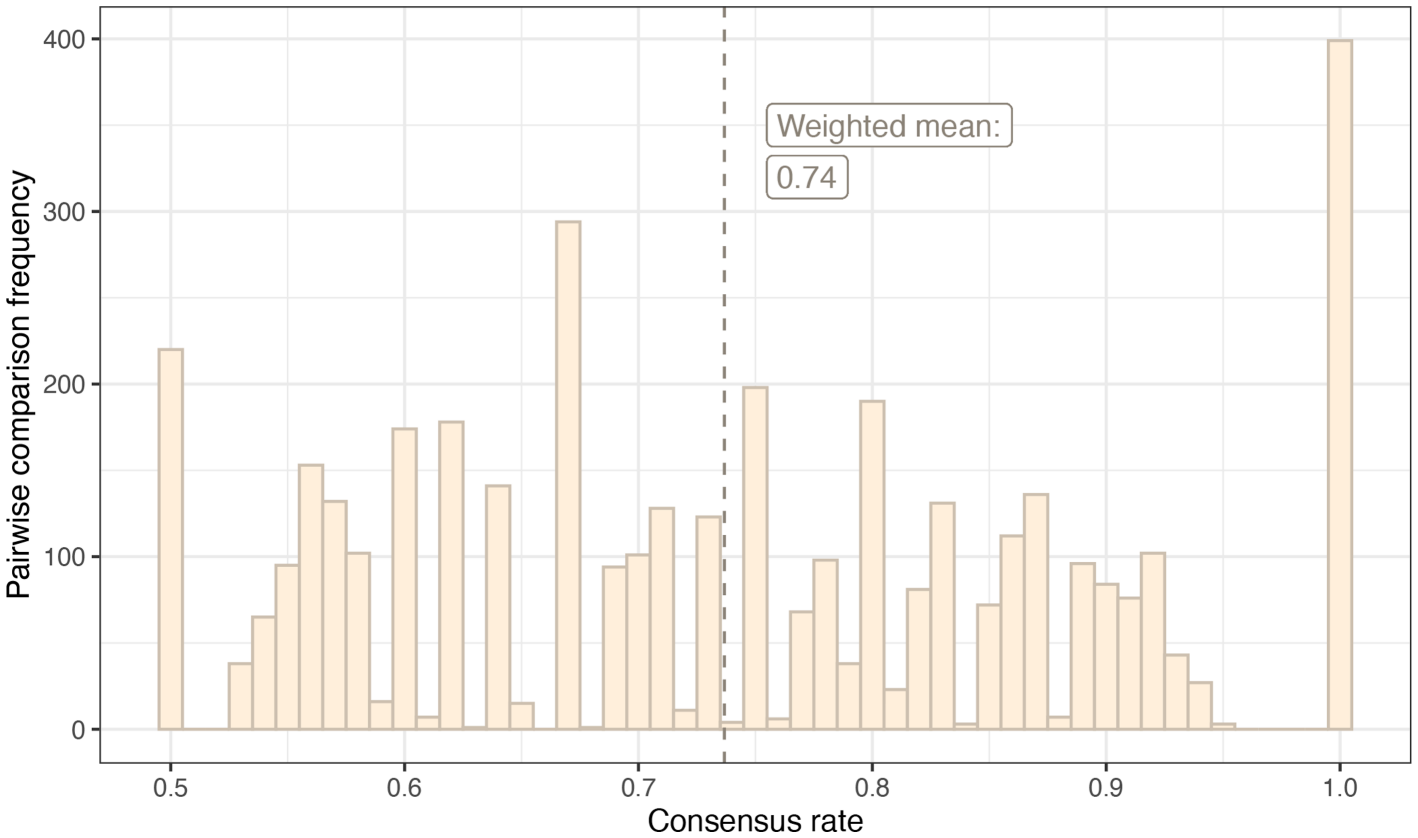
A histogram demonstrating the frequency of pairwise comparisons per consensus rate in the main survey.

In the reliability test, the weighted mean consensus rate was 0.76. This was consistent with the weighted mean consensus rate in the main survey: 0.74. The distribution of consensus rates across applicable pairwise comparisons in the main survey is shown in Figure 5.

Individually, each reliability metric was compared to the distribution of the same metric produced by 5,000 simulated, random responses to the reliability test (Table 3). All mean (or median) reliability metrics of the random samples were significantly less than the test metrics produced by the real respondents at the *p* < 0.01 level.

**Table 3:** Inter and intra-rater reliability metrics compared to simulated, random responses **p ≤ 0.01.

		Symmetry consistency	Triplet transitivity	Test re-test stability	Inter-rater reliability (weighted mean consensus rate, WMCR)
Metric from the reliability test data (test metric)		0.92	0.97	0.74	0.76
Simulated, random response data	Method	5,000 random responses to the reliability test were produced and the proportion of instances where responses to symmetrical questions (A vs. B and B vs. A) were equal, per simulated respondent, were calculated	5,000 random responses to the reliability test were produced and the triplet transitivity rates, per simulated respondent, were calculated	5,000 random responses to the reliability test were produced, and then repeated. The test re-test stability of the responses in the two sets was calculated. This was repeated 4,999 more times, each time comparing the new set of responses to the first iteration	5,000 random responses to the reliability test were produced and the WMCR calculated. This was repeated a further 4,999 times, with a new set of random responses each time
	Metric mean/median (range)	0.538 (median) (0–1.000) *n* = 5,000	0.769 (median) (0.231–1.000) *n* = 5,000	0.500 (mean) (0.497–0.502) *n* = 5,000	0.506 (mean) (0.504–0.508) *n* = 5,000
Hypothesis test	Test	Wilcoxon one sample median test (simulated data is ordinal)	Wilcoxon one sample median test (simulated data is ordinal)	One sample *t*-test (simulated data is approximately normal on QQ plot examination)	One sample *t*-test (simulated data is approximately normal on QQ plot examination)
	H_1_	The median symmetry consistency metric of the simulated, random response sample is less than the test metric	The median triplet transitivity rate of the simulated, random response sample is less than the test metric	The mean test–retest stability metric of the simulated, random response samples is less than the test metric	The mean MWCR of the simulated, random response sample is less than the test metric
	*p*-value	*p* ≤ 2.2e–16**	*p* ≤ 2.2e–16**	*p* ≤ 2.2e–16**	*p* ≤ 2.2e–16**

#### 4.5.3. Consistency indices

**Figure 6:**
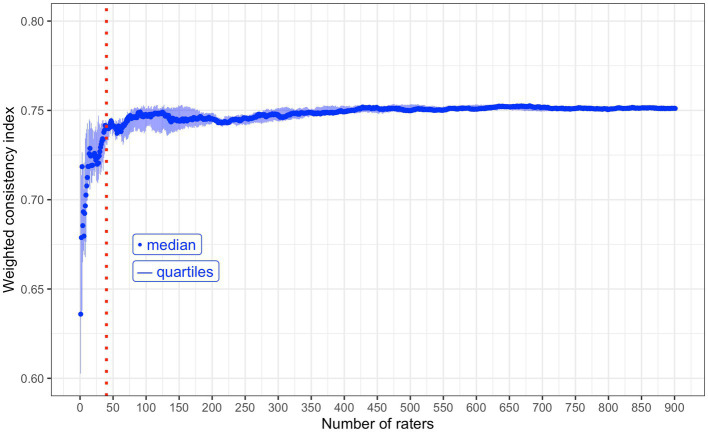
A chart to demonstrate the median weighted consistency index with increasing numbers of raters included in the calculation. This includes both student and clinical supervisor raters together. Red, dotted, vertical reference line: x = 40 raters. Forty is the minimum number of raters aimed for. This value was calculated using data taken from raters of a single demographic group (Supplementary material 2).

In the main survey, the non-weighted consistency index was 0.67; in about two of every three instances, the pairwise comparison result went in the direction of the expectation according to the running Elo ratings of the items in question. The mean weighted consistency index (weighted for the surprisingness of an upset result) for the main survey was 0.75. The weighted consistency index was calculated with increasing numbers of raters included. The consistency index quickly stabilised at around 0.75 when approximately 75 raters (students and clinical supervisors) were included in the calculation (Figure 6).

#### 4.5.4. Method reproducibility

The Pearson’s and Kendall’s Tau correlation coefficients between the n.mElo ratings and rankings generated by two halves of a single demographic group’s dataset was *r*(89) = 0.92, *p* ≤ 2.2e–16 (very strong) and *r*_τ_(89) = 0.75, *p* ≤ 2.2e–16 (strong), respectively (58, 59).

## 5. Discussion

This methods paper has demonstrated that using pairwise comparison data, the Elo algorithm can generate relative ratings and rankings of a large number of entities on a single scale. In the example provided, preparedness characteristics were rated in terms of their relative importance for veterinary WCT, according to the perspectives of a group of veterinary students and clinical workplace supervisors. The output of the algorithm is a vector of ratings taking values between zero and one. The values represent the relative degree of the measured criterion (e.g., importance) attributed to each entity. When *n* ratings are sorted in order of descending value they can be converted to a ranking vector, taking values from one to *n*.

This method could be readily adapted by other researchers to quantitatively measure perspectives when there is a large number of entities to compare on a relative scale. These ratings provide a different perspective to the absolute ratings provided by other systems such as Likert items. Future applications of this methodology could include academics comparing written essays to form a relative rating and ranking of students’ performance (41, 60), students comparing clinical competencies for their ease of acquisition to form a relative rating and ranking from easy to hard, or students comparing study tools in terms of their frequency of use to form a relative rating and ranking from most to least utilised. The data set produced by this method will provide unique insights for HPE researchers, but it can also be used to triangulate other findings such as qualitative or other survey data.

Beyond pairwise comparison questions, there are other question types available when challenged with measuring attitudes about a large number of entities. Alternatives include using visual analogue scales, which are essentially Likert items on a graphic line. This question style might produce more granular data than Likert items, but they are also likely to be susceptible to the ceiling effect and greater measurement error. Best-worst scaling measurements (61) could also be considered. However, best-worst scaling requires participants to consider their attitude of more than two entities at once, which has a higher cognitive load than simply comparing two. Keeping the comparison free from extraneous influences caused by the presence of other entities or dimensions is likely to reduce measurement error and increase precision (23).

### 5.1. Survey metadata, validity and reliability

There is evidence that long online surveys are associated with higher non-response rates (62–64), higher proportions of “do not know” answers, and higher semi-completion (64). This was of particular concern when designing a method to rate a large set of items because rating each one individually was likely to be a lengthy process. There is evidence that the ideal length of an online survey is between 10 and 15 minutes (65, 66), and for this pairwise comparison-based survey, the median time to complete was 14.1 minutes which sits in this bracket.

Except for a single outlier respondent who selected “I do not understand one or both of the options” almost 50% of the time (Figure 3), there is little evidence for systematic responding in the pairwise comparisons. This is supported by the fact that each of the two options (the item listed first, or the one listed second) were selected strikingly close to half of the time (51.4 and 48.3%, respectively). This outcome is facilitated by the fact that both question order and response option order were randomised on the survey delivery platform.

Using qualitative data from relevant group interviews to generate the survey’s items (preparedness characteristics) meant that there was inherent validity to the survey content. The interviews reached “data sufficiency” (67, 68) which supports the comprehensiveness of the list of preparedness characteristics used. Qualitative validity was supported by *post-hoc* objective content validity indices (CVIs). Less than 20% of preparedness characteristics had an average CVI equivalent to not (*n* = 4) or somewhat (*n* = 13) relevant to the survey and removing these items from the survey could be justified. Further validity evidence could be sought, for example by use of a Delphi method (43), to determine whether these, or any other, items should be removed from the set, or, indeed, if any that are missing should be added.

The reliability test data demonstrated that pairwise comparison style questions can be answered reliably by a single rater (symmetry consistency = 0.92 and triplet transitivity rate = 0.97). The test–retest reliability was lower than these other measurements of intra-rater reliability. This is to be expected given that the intra-rater reliability measures calculated solely from responses in the first sitting benefit from respondents’ short-term memory, and respondents are typically reluctant to give inconsistent responses (8).

In the reliability test and the main survey, the weighted mean consensus rates were consistent at 0.76 and 0.74, respectively. This suggests that inter-rater reliability is lower than the intra-rater reliability, which is to be expected when examining group perspectives on a subjective topic because there is likely to be variation between individuals. One of the elegancies of the Elo algorithm is that all participants’ perspectives are taken on board to generate a mathematical consensus (the Elo rating). Just as sports team A might not beat sports team B every time they play, if sufficient matches are played then the ranking of team A will consistently be higher than B, despite sometimes losing. In this study, sufficient ‘matches’ were played (or pairwise comparisons performed/raters included), because the weighted consistency index plateaued with around 75 raters’ pairwise comparison data included, yet data from 901 raters was collected. Clark et al. state that the number of raters included at the point of the index plateau can be interpreted as the minimum number of participants required to generate a stable ranking of Elo ratings in that specific setting (19). In other scenarios, this minimum is likely to change in line with the inter- and intra-rater consistency in responses. As an example, the same procedure using data from a single demographic group (students only, as opposed to students and supervisors here) plateaued at *n* = 20 (Supplementary material 2), which is similar to the number of raters required by Clark et al. (*n* = 30–40) (19).

To summarise, respondents were able to answer the pairwise comparison questions reliably, in a reasonable amount of time and without introducing systematic bias. Although inter-rater reliability was lower than intra-rater reliability, we have demonstrated that when enough raters complete the survey, the Elo algorithm is able to produce stable rankings of the items in question. The number of raters required is likely to depend on the diversity of opinion within the group. Reviewing the feedback from multiple validity approaches provided reasonable assurance that the survey is able to achieve the study objective.

### 5.2. Lessons learnt from the application of pairwise comparison questions and the Elo algorithm

Stemming from Thurstone’s law of comparative judgement (69, 70), the assumption that participants can detect even the smallest difference in their preference when performing comparisons has been questioned (71). Alternatively, there is probably a threshold level of difference that must be exceeded for a judgement to be made. Considering the survey design, one resolution could be to add an indifference option for participants, in case they are unable to distinguish between the items. However, it is anticipated that a larger volume of data (more pairwise comparisons per participant, or more participants) would be required to generate stable ratings. Additionally, even if the pairwise comparisons containing very similar items are answered inconsistently, the items will still form a stable ranking because they will be compared with many other items too. When imperfect judgements are aggregated, the collective intelligence can be excellent; the key to solving a problem is not always to summon the expert, but to ask the crowd instead (72). This is a distinction of the Elo system; the rating and ranking of an item is not informed by only one piece of information (a rating of the item itself by individual participants), but many pieces (serial comparisons of the item to several others, by a group of participants).

In addition to the challenge of rating very similar items, it is acknowledged that it may be difficult for participants to make a choice because some comparisons may not feel natural to make. In this specific use case, an example might be choosing between a relatively abstract characteristic such as “an awareness of the complex professional and culture norms of the veterinary workplace” with a characteristic that is very specific and tangible such as “knowledge of the core vaccines for domestic species.” This challenge was acknowledged and explained to participants in the information provided prior to commencing the survey. The items could have been broken down into separate domains such as knowledge, skills, personal attributes, behaviours, and only within-domain comparisons offered to participants. However, this would produce importance ratings for items relative to other items in that domain only, greatly reducing the impact of the method. It may be that in other applications of this method, where items are more uniform and obviously directly comparable, the problem of awkward comparisons will not be significant.

Another potential challenge is related to question precision; ensuring that all items mean the same thing to all participants, although this is not unique to pairwise comparisons alone. Cognitive interviews (73) during the validation phase of survey development could have been useful to ensure consistent question interpretation. To partially address it, a dictionary was produced to provide participants with more detailed descriptions of the characteristics, which could be referred to at any point. This was in an attempt to unify the understanding of the characteristics, but there is no guarantee that it was used or that it increased the precision of the items. If, after consulting the dictionary, participants still did not understand any item, they were able to select a third option “I do not understand one or both of the options.” These outcomes were removed prior to entry into the Elo algorithm which can only handle binary winner/loser data. This was preferable to the participant guessing which characteristic they think is more important, which would introduce measurement error.

### 5.3. Limitations of pairwise comparison questions and the Elo algorithm

This method requires an online platform to host the survey, delivering random pairwise comparisons from a question bank. The authors used Qualtrics^6^ which is a paid-for service approved by the institution for use in research. However, this platform (or one with similar features) may not be available to all HPE researchers, which is a limitation. Pairwise comparisons are fundamentally multiple-choice questions and can be produced using free services, such as Google Forms,^7^ however administering a unique, random subset to each participant may be more challenging on these platforms.

The ratings generated from pairwise comparison data and the Elo algorithm cannot be attributed to a label. As an example in this study, the n.mElo ratings equate the most important to the least important preparedness characteristics, but that is not to say that the least important item is not important at all. Here, this is an advantage as the items have already been identified as somewhat important by group interview participants and we are interested in their relative importance.

**Figure 7:**
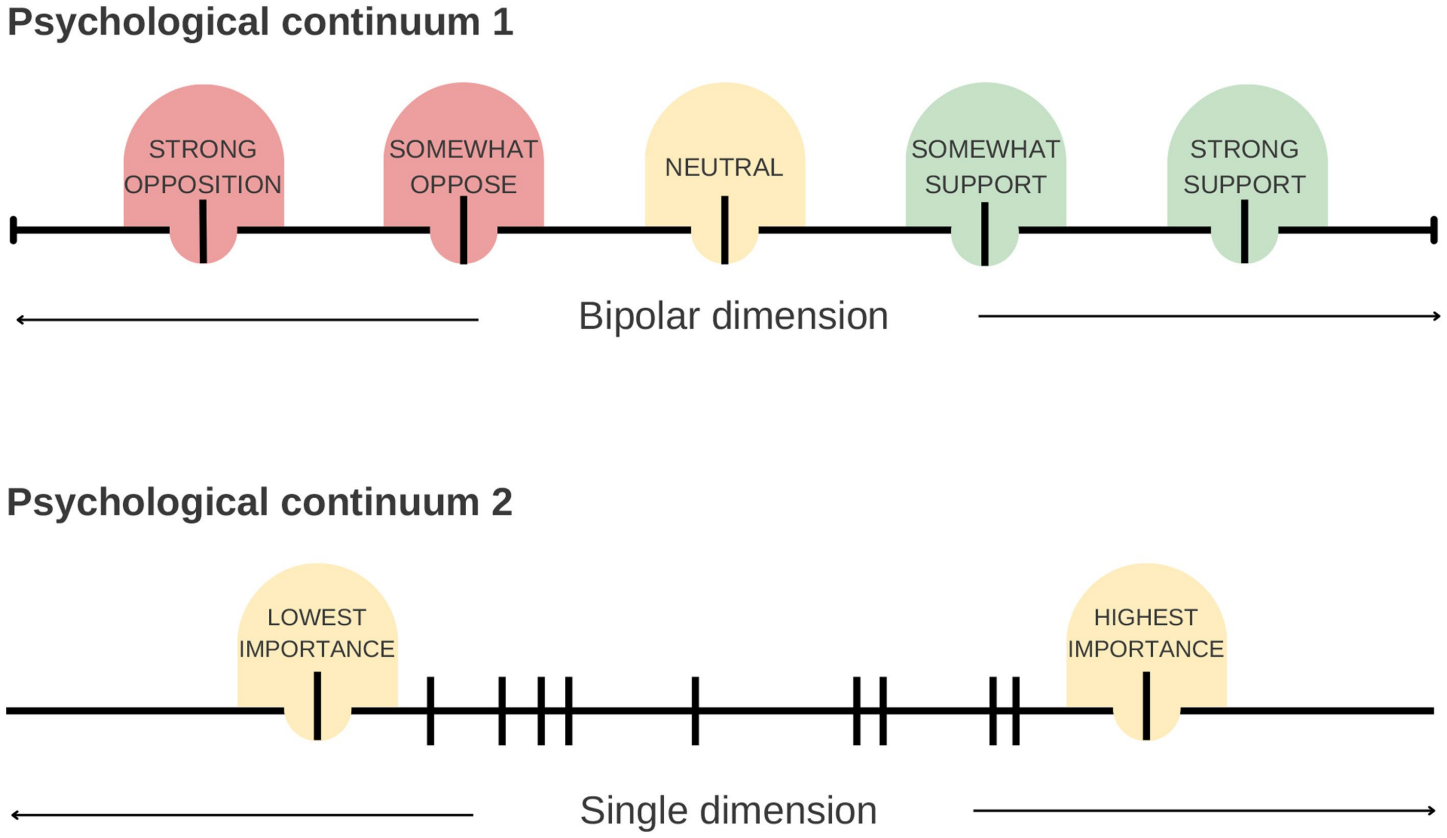
Two example psychological continua that could be examined using a survey in health professions education (HPE) research. As in the case study of this paper, the “importance” of different entities can be considered in a single dimension, with the importance of measured entities as relative to each other. This is well suited to measurement using pairwise comparison questions and the Elo algorithm. Some researched dimensions, though, are bidirectional and span from one extreme (e.g., opposition) to the other (e.g., support). Measurement of different entities on this scale is better suited to Likert items, which can measure both aspects of the dimension absolutely. In contrast, the outcomes of pairwise comparisons measuring “support” would not measure active opposition, only relatively less support.

However, this output format could be viewed as a limitation in studies where an absolute (not relative) measure is required, or the entities could theoretically be attributed bivalent labels (opposite criteria). As an example, if an HPE researcher’s aim was to determine the acceptability of potential new teaching interventions, their objective could be to measure perspectives on the interventions on an absolute scale, ranging from totally unacceptable to perfectly acceptable. The pairwise comparison method would generate ratings indicating the relatively least to most acceptable items. However, this output may not align with the research aim since it fails to capture whether students perceive the “least acceptable” item as truly unacceptable. This demonstrates the nuanced distinction between the two output formats. This research example would be a case where using Likert items to generate absolute ratings would be useful. In general, pairwise comparisons and the Elo algorithm are probably best suited for *comparing* items in a single dimension (e.g., the item is higher or lower priority) whereas Likert items are probably best suited when an absolute measurement across a wide spectrum is required (e.g., the item has a negative effect on X to a positive effect on X) (Figure 7). Therefore, the suitability of pairwise comparisons to generate ratings for survey items must be carefully considered in light of the research question at hand.

Generally, the Elo algorithm cannot produce item ratings on a per participant basis. The input data for the Elo algorithm are the pooled pairwise comparisons outcomes from any group of interest and the output is the n.mElo ratings of the items according to the perspectives of the group. It is useful to consider the survey participants as working as a hivemind, each contributing their own share of the input data. An exception to this would be if each participant performed a sufficient number of pairwise comparisons, likely hundreds, to generate sufficiently stable rankings of n.mElo ratings per person. This limitation is problematic if the researcher’s aims included understanding perspectives on an individual basis, but this was not required in this study.

Frequently HPE researchers seek to explore differences in perspectives *between* groups of survey respondents. For example, do students and workplace supervisors perceive different preparedness characteristics to be relatively more important? Generating n.mElo ratings on a per group basis impacts the statistical techniques used for between-group comparisons. Traditional hypothesis testing tends to focus on determining whether the average rating of an item from two groups are significantly different in location whilst considering the variability of the ratings in the groups. Since n.mElo ratings are not produced on a per participant basis, and there is only a single value of n.mElo for the group, this variation is not “known” or “produced” by the algorithm. There are ways to circumvent this issue (see 5.4 Further work).

### 5.4. Further work

Correlation analysis can be used to determine whether the n.mElo ratings and rankings according to different groups are different. However, further data processing and analysis is required in order to determine for *which* items the n.mElo ratings are different. This includes bootstrapping (74) pairwise comparisons to generate 95% confidence intervals for the n.mElo ratings (or rankings). The next step would be to discern whether there is overlap in the confidence intervals for an item between each group. Alternatively, a 95% confidence interval for the *difference* in n.mElo ratings (or rankings) of an item between groups can be generated and it should be examined for whether it includes zero. Both of these outcomes would signify that the n.mElo ratings (or rankings) for the item are significantly different between groups at the 0.05 level.

A complementary method would be to use permutation tests (74, 75). The permutation test is a hypothesis test which builds, rather than assumes, a null distribution by resampling the observed data (pairwise comparisons) without replacement. The observed test statistic (the true difference in n.mElo ratings (or rankings) of an item between groups) is subsequently compared to this distribution for its extremeness. The p-value represents the probability of obtaining the test statistic assuming that the null distribution is true and provides a more exact indication of whether the n.mElo ratings (or rankings) for an item are significantly different between groups.

If the survey is applied at two time points instead of to different participant groups, bootstrapping to generate confidence intervals and permutation tests could also be used to map how attitudes and ratings change over time. As an example, to determine if students’ perceptions on the importance of different preparedness characteristics changes as they experience their training or as part of programmatic assessment to monitor student progress.

Criterion validity [how well a test correlates with a different measure (76)] could be assessed by correlating n.mElo ratings with Likert item ratings. Although it should be acknowledged these methods would measure different aspects of the attribute (relative versus absolute), if n.mElo ratings are a valid indicator of a construct of interest, then one would expect the characteristics with the highest n.mElo ratings to also have the highest Likert item ratings, and vice versa. Additionally, comparing the two systems would provide further information about how both methods react to the measurement of perceptions about a particularly large number of entities (e.g., response times).

Further work to verify the stability of items’ ranks could be performed. This could involve repeatedly running the Elo algorithm, starting with one participant’s pairwise comparison data and each time increasing the number of participants included by one. The *change* in the rank of each item as another participant’s data is added would be calculated in addition to the mean change across the set of items. The mean change in rank is expected to decrease exponentially and approach zero as increasing numbers of participants take part.

### 5.5. Conclusion

The method presented in this paper is a useful option for HPE researchers seeking to measure relative perspectives across a range of items. Use cases might include prioritising clinical skills for inclusion in an HPE curriculum, or assessing which challenges associated with work placements pose a larger problem for students. Given the frequency with which surveys are used in HPE research to assess a wide range of research topics, this novel approach has the potential to be highly impactful.

The method consists of two stages. Firstly, a survey comprising pairwise comparison questions task research participants with assessing items in pairs, in terms of a single quality, feature, or attribute. Subsequently the Elo rating system processes the survey data to generate relative ratings and rankings of the items on a unidimensional scale of the attribute.

## Data availability statement

The original contributions presented in the study are included in the article/Supplementary material, further inquiries can be directed to the corresponding author.

## Ethics statement

The studies involving human participants were reviewed and approved by the University of Surrey Ethics Committee (FHMS 20-21 118 EGA Amend 2 04/03/22). The patients/participants provided their written informed consent to participate in this study.

## Author contributions

KJ devised the project and secured funding for the research, which is supervised by KJ, SP, and PC. JRo conceived the main conceptual idea of this manuscript, performed the data analysis, and prepared the draft manuscripts which all authors contributed to editing. JRo, KJ, SP, and PC developed the study design and the survey. SWo, SWa, JRe, CW, AR, and PP significantly contributed to data collection (recruitment of student participants and completing the reliability and validity tests). All authors contributed to the article and approved the submitted version.

## Funding

JRo is funded by the Longhurst Legacy at the University of Surrey.

## Conflict of interest

The authors declare that the research was conducted in the absence of any commercial or financial relationships that could be construed as a potential conflict of interest.

## Publisher’s note

All claims expressed in this article are solely those of the authors and do not necessarily represent those of their affiliated organizations, or those of the publisher, the editors and the reviewers. Any product that may be evaluated in this article, or claim that may be made by its manufacturer, is not guaranteed or endorsed by the publisher.

## Footnotes

^1^RStudio, 250 Northern Ave, Boston, MA 02210, United States https://www.rstudio.com

^2^ https://github.com/jennyrouth/n.mEloRatings

^3^ https://CRAN.R-project.org/package=EloChoice

^4^Qualtrics XM, 10 York Road, London SE1 7ND, United Kingdom https://www.qualtrics.com/

^5^ https://bit.ly/optionhelpsheet

^6^ https://www.qualtrics.com/uk/

^7^ https://www.google.co.uk/forms/about/

## References

[1] Artino ARJ Phillips AW Utrankar A Ta AQ Durning SJ. “The questions shape the answers”: assessing the quality of published survey instruments in health professions education research. Acad Med. (2018) 93:456–63. doi: 10.1097/ACM.0000000000002002 29095172

[2] Phillips AW Friedman BT Utrankar A Ta AQ Reddy ST Durning SJ. Surveys of health professions trainees: prevalence, response rates, and predictive factors to guide researchers. Acad Med. (2017) 92:222–8. doi: 10.1097/ACM.0000000000001334 27532869

[3] Artino AR la Rochelle JS Dezee KJ Gehlbach H. Developing questionnaires for educational research: AMEE guide no. 87. Med Teach. (2014) 36:463–74. doi: 10.3109/0142159X.2014.889814, PMID: 24661014 PMC4059192

[4] Carifio J Perla RJ. Ten common misunderstandings, misconceptions, persistent myths and urban legends about Likert scales and Likert response formats and their antidotes. J Soc Sci. (2007) 3:106–16. doi: 10.3844/jssp.2007.106.116

[5] Uebersax J . Likert Scales: Dispelling the Confusion. Statistical Methods for Rater Agreement. (2006). Available at: http://john-uebersax.com/stat/likert.htm. (Accessed October 26, 2022).

[6] Saadeh K Aitken JB Paramasivam SJ Cockcroft P Jeevaratnam K. Student perspectives of preparedness characteristics for clinical learning within a fully distributed veterinary teaching model. PLoS One. (2021) 16:e0249669. doi: 10.1371/journal.pone.0249669, PMID: 33983962 PMC8118455

[7] Voutilainen A Pitkäaho T Kvist T Vehviläinen-Julkunen K. How to ask about patient satisfaction? The visual analogue scale is less vulnerable to confounding factors and ceiling effect than a symmetric Likert scale. J Adv Nurs. (2016) 72:946–57. doi: 10.1111/jan.12875, PMID: 26689434

[8] Tourangeau R Rips LJ Rasinski K. The Psychology of Survey Response. 1st ed. Cambridge, UK: Cambridge University Press (2000).

[9] Cohen L Manion L Morrison K. Research Methods in Education. 8th ed. Abingdon, Oxfordshire: Routledge (2017).

[10] Vogt PW . Dictionary of Statistics & Methodology. 3rd ed. London: SAGE Publications Inc (2005).

[11] Brown DR . Stimulus-similarity and the anchoring of subjective scales. Am J Psychol. (1953) 66:199–214. doi: 10.2307/1418726, PMID: 13040526

[12] Wedell DH Parducci A Geiselman RE. A formal analysis of ratings of physical attractiveness: successive contrast and simultaneous assimilation. J Exp Soc Psychol. (1987) 23:230–49. doi: 10.1016/0022-1031(87)90034-5

[13] Manis M Biernat M Nelson TF. Comparison and expectancy processes in human judgment. J Pers Soc Psychol. (1991) 61:203–11. doi: 10.1037/0022-3514.61.2.203 1920062

[14] Schwarz N Bles H. Scandals and the Public's Trust in Politicians: assimilation and contrast effects. Personal Soc Psychol Bull. (1992) 18:574–9. doi: 10.1177/0146167292185007

[15] Groves RM Fowler FJ Jr Couper MP Lepkowski JM Singer E Tourangeau R. Survey Methodology. 2nd ed. Hoboken: Wiley (2009).

[16] Johnson MD Lehmann DR Horne DR. The effects of fatigue on judgments of interproduct similarity. Int J Res Mark. (1990) 7:35–43. doi: 10.1016/0167-8116(90)90030-Q

[17] Krosnick JA . Response strategies for coping with the cognitive demands of attitude measures in surveys. Appl Cogn Psychol. (1991) 5:213–36. doi: 10.1002/acp.2350050305

[18] Krosnick JA Berent MK. Comparisons of party identification and policy preferences: the impact of survey question format. Am J Polit Sci. (1993) 37:941–64. doi: 10.2307/2111580

[19] Clark AP Howard KL Woods AT Penton-Voak IS Neumann C. Why rate when you could compare? Using the "EloChoice" package to assess pairwise comparisons of perceived physical strength. PLoS One. (2018) 13:e0190393. doi: 10.1371/journal.pone.0190393 29293615 PMC5749798

[20] Wolfert P Girard JM Kucherenko T Belpaeme T, (Eds.) To Rate or Not to Rate: Investigating Evaluation Methods for Generated Co-speech Gestures. Proceedings of the 2021 International Conference on Multimodal Interaction; (2021).

[21] Phelps AS Naeger DM Courtier JL Lambert JW Marcovici PA Villanueva-Meyer JE . Pairwise comparison versus Likert scale for biomedical image assessment. Am J Roentgenol. (2014) 204:8–14. doi: 10.2214/AJR.14.13022 25539230

[22] Jamieson S . Likert scales: how to (ab)use them. Med Educ. (2004) 38:1217–8. doi: 10.1111/j.1365-2929.2004.02012.x, PMID: 15566531

[23] David HA . The Method of Paired Comparisons. 2nd ed. London: Griffin (1988). 188 p.

[24] Elo AE . The Rating of Chessplayers, Past and Present. New York: Arco Pub. (1978).

[25] Langville AN Meyer CD. Who’s #1? The Science of Rating and Ranking. Oxford, UK: Princeton University Press (2012).

[26] Elliott LL . Reliability of judgments of figural complexity. J Exp Psychol. (1958) 56:335–8. doi: 10.1037/h0043971, PMID: 13587863

[27] Mueser KT Grau BW Sussman S Rosen AJ. You're only as pretty as you feel: facial expression as a determinant of physical attractiveness. J Pers Soc Psychol. (1984) 46:469–78. doi: 10.1037/0022-3514.46.2.469

[28] Kułakowski K . Inconsistency in the ordinal pairwise comparisons method with and without ties. Eur J Oper Res. (2018) 270:314–27. doi: 10.1016/j.ejor.2018.03.024

[29] Luce RD . Response Times: Their Role in Inferring Elementary Mental Organization. New Edition ed. New York: Oxford University Press (1991).

[30] Massey K . Statistical Models Applied to the Rating of Sports Teams. Bluefield, VA, USA: Bluefield College (1997).

[31] Colley WN . Colley Matrix Colley’s Bias Free Matrix Rankings; (2002). Available at: https://www.colleyrankings.com. (Accessed September 23, 2021).

[32] Keener JP . The Perron–Frobenius theorem and the ranking of football teams. SIAM Rev. (1993) 35:80–93. doi: 10.1137/1035004

[33] Bradley RA Terry ME. Rank analysis of incomplete block designs: I. the method of paired comparisons. Biometrika. (1952) 39:324–45.

[34] Saaty TL . The Analytic Hierarchy Process. New York: McGraw Hill (1980).

[35] Silver N Boice J Paine N. How Our NFL Predictions Work; (2019). Available at: https://fivethirtyeight.com/methodology/how-our-nfl-predictions-work/. (Accessed May 20, 2021).

[36] Pieramati C Fusaioli L Scacco L Buttazzoni L Silvestrelli M. On the use of elo rating on harness racing results in the genetic evaluation of trotter. Ital J Anim Sci. (2007) 6:189–91. doi: 10.4081/ijas.2007.1s.189

[37] Herbrich R Minka T Graepel T, (Eds.) TrueSkill™: A Bayesian Skill Rating System. Proceedings of the 19th International Conference on Neural Information Processing Systems; (2006).

[38] Newton-fisher NE . Modeling social dominance: Elo-ratings, prior history, and the intensity of aggression. Int J Primatol. (2017) 38:427–47. doi: 10.1007/s10764-017-9952-2, PMID: 28680188 PMC5487812

[39] Goodspeed R . Research note: an evaluation of the Elo algorithm for pairwise visual assessment surveys. Landsc Urban Plan. (2017) 157:131–7. doi: 10.1016/j.landurbplan.2016.06.009

[40] Krüger S Charlotte SA. Judging books by their covers – tinder interface, usage and sociocultural implications. Inf Commun Soc. (2020) 23:1395–410. doi: 10.1080/1369118X.2019.1572771

[41] Pelánek R . Applications of the Elo rating system in adaptive educational systems. Comput Educ. (2016) 98:169–79. doi: 10.1016/j.compedu.2016.03.017

[42] Trevitt CMNRN Grealish LMNRN Reaby LPRN. Students in transit: using a self-directed preceptorship package to smooth the journey. J Nurs Educ. (2001) 40:225–8. doi: 10.3928/0148-4834-20010501-09, PMID: 11355762

[43] Chipchase LS Buttrum PJ Dunwoodie R Hill AE Mandrusiak A Moran M. Characteristics of student preparedness for clinical learning: clinical educator perspectives using the Delphi approach. BMC Med Educ. (2012) 12:112. doi: 10.1186/1472-6920-12-112, PMID: 23145840 PMC3527360

[44] Spiliotopoulou G . Preparing occupational therapy students for practice placements: initial evidence. Br J Occup Ther. (2007) 70:384–8. doi: 10.1177/030802260707000903

[45] Virtue SM Pendergast L Tellez M Waldron E Ismail A. Identifying noncognitive skills that contribute to dental students’ success: dental faculty perspectives. J Dent Educ. (2017) 81:300–9. doi: 10.1002/j.0022-0337.2017.81.3.tb06275.x, PMID: 28250036

[46] Morrell N Ridgway V. Are we preparing student nurses for final practice placement? Br J Nurs. (2014) 23:518–23. doi: 10.12968/bjon.2014.23.10.518 24851915

[47] Sturman N Régo P Dick M-L. Rewards, costs and challenges: the general practitioner’s experience of teaching medical students. Med Educ. (2011) 45:722–30. doi: 10.1111/j.1365-2923.2011.03930.x, PMID: 21649705

[48] Cake MA Bell MA Williams JC Brown FJL Dozier M Rhind SM . Which professional (non-technical) competencies are most important to the success of graduate veterinarians? A best evidence medical education (BEME) systematic review: BEME guide no. 38. Med Teach. (2016) 38:550–63. doi: 10.3109/0142159X.2016.1173662, PMID: 27145182

[49] Banneheke H Nadarajah VD Ramamurthy S Sumera A Ravindranath S Jeevaratnam K . Student preparedness characteristics important for clinical learning: perspectives of supervisors from medicine, pharmacy and nursing. BMC Med Educ. (2017) 17:130. doi: 10.1186/s12909-017-0966-4, PMID: 28789645 PMC5549327

[50] Judd B Brentnall J Scanlan JN Thomson K Blackstock F Mandrusiak A . Evaluating allied health students’ readiness for placement learning. BMC Med Educ. (2023) 23:70. doi: 10.1186/s12909-023-04005-w, PMID: 36709272 PMC9883866

[51] Routh J Paramasivam SJ Cockcroft P Nadarajah VD Jeevaratnam K. Stakeholder perspectives on veterinary student preparedness for workplace clinical training – a qualitative study. BMC Vet Res. (2022) 18:340. doi: 10.1186/s12917-022-03439-6, PMID: 36085152 PMC9461096

[52] Guetterman TC Fetters MD Creswell JW. Integrating quantitative and qualitative results in health science mixed methods research through joint displays. Ann Fam Med. (2015) 13:554–61. doi: 10.1370/afm.1865, PMID: 26553895 PMC4639381

[53] Han J Kamber M Pei J. Data preprocessing In: Han J Kamber M Pei J, editors. Data Mining: Concepts and Techniques. 3rd ed. Boston: Morgan Kaufmann (2012). 83–124.

[54] Mokkink LB Terwee CB Patrick DL Alonso J Stratford PW Knol DL . The COSMIN study reached international consensus on taxonomy, terminology, and definitions of measurement properties for health-related patient-reported outcomes. J Clin Epidemiol. (2010) 63:737–45. doi: 10.1016/j.jclinepi.2010.02.006, PMID: 20494804

[55] Polit DF Yang FM. Measurement and the Measurement of Change: A Primer for the Health Professions. Wolters Kluwer: Philaelphia, PA (2016).

[56] Zhou Y . A mixed methods model of scale development and validation analysis. Meas Interdiscip Res Perspect. (2019) 17:38–47. doi: 10.1080/15366367.2018.1479088

[57] Polit DF Beck CT Owen SV. Is the CVI an acceptable indicator of content validity? Appraisal and recommendations. Res Nurs Health. (2007) 30:459–67. doi: 10.1002/nur.20199, PMID: 17654487

[58] Schober P Boer C Schwarte LA. Correlation coefficients: appropriate use and interpretation. Anesth Analg. (2018) 126:1763–8. doi: 10.1213/ANE.0000000000002864 29481436

[59] Botsch R . Chapter 12: Significance and Measures of Association. Scopes and Methods of Political Science; (2011). Available at: http://polisci.usca.edu/apls301/Text/Chapter%2012.%20Significance%20and%20Measures%20of%20Association.htm. (Accessed February 22, 2023).

[60] Gray A Rahat A Crick T Lindsay S Wallace D. Using Elo rating as a metric for comparative judgement in educational assessment. Proceedings of the 6th International Conference on Education and Multimedia Technology. Guangzhou, China. (2022).

[61] Burton N Burton M Rigby D Sutherland CAM Rhodes G. Best-worst scaling improves measurement of first impressions. Cogn Res Princ Implic. (2019) 4:36. doi: 10.1186/s41235-019-0183-2, PMID: 31549257 PMC6757072

[62] Crawford SD Couper MP Lamias MJ. Web surveys:perceptions of burden. Soc Sci Comput Rev. (2001) 19:146–62. doi: 10.1177/089443930101900202

[63] Marcus B Bosnjak M Lindner S Pilischenko S Schütz A. Compensating for low topic interest and long surveys:a field experiment on nonresponse in web surveys. Soc Sci Comput Rev. (2007) 25:372–83. doi: 10.1177/0894439307297606

[64] Deutskens E de Ruyter K Wetzels M Oosterveld P. Response rate and response quality of internet-based surveys: an experimental study. Mark Lett. (2004) 15:21–36. doi: 10.1023/B:MARK.0000021968.86465.00

[65] Revilla M Höhne JK. How long do respondents think online surveys should be? New evidence from two online panels in Germany. Int J Mark Res. (2020) 62:538–45. doi: 10.1177/1470785320943049

[66] Revilla M Ochoa C. Ideal and maximum length for a web survey. Int J Mark Res. (2017) 59:557–65. doi: 10.2501/IJMR-2017-039

[67] Varpio L Ajjawi R Monrouxe LV O'Brien BC Rees CE. Shedding the cobra effect: problematising thematic emergence, triangulation, saturation and member checking. Med Educ. (2017) 51:40–50. doi: 10.1111/medu.13124, PMID: 27981658

[68] Dey I . Grounding Grounded Theory: Guidelines for Qualitative Inquiry. London: Academic Press (1999). 115–48.

[69] Thurstone LL . A law of comparative judgment. Psychol Rev. (1927) 34:273–86. doi: 10.1037/h0070288

[70] Thurstone LL . The method of paired comparisons for social values. J Abnorm Soc Psychol. (1927) 21:384–400. doi: 10.1037/h0065439

[71] Falmagne J-C . Elements of Psychophysical Theory. Cary, USA: Oxford University Press Inc. (1985).

[72] Surowiecki J . The Wisdom of Crowds. New York, USA: Anchor Books (2005).

[73] Drennan J . Cognitive interviewing: verbal data in the design and pretesting of questionnaires. J Adv Nurs. (2003) 42:57–63. doi: 10.1046/j.1365-2648.2003.02579.x, PMID: 12641812

[74] Efron B Tibshirani RJ. An Introduction to the Bootstrap. Dordrecht: Springer - Science + Business Media (1993).

[75] Wilber J . The Permutation Test a Visual Explanation of Statistical Testing; (2019). Available at: https://www.jwilber.me/permutationtest/. (Accessed November 07, 2022).

[76] Lohmeier JH . Criterion-based validity evidence In: Frey BB , editor. The SAGE Encyclopedia of Educational Research, Measurement, and Evaluation. Thousand Oaks, California: SAGE Publications, Inc. (2018)

