# Supplementary material for: Rating and ranking preparedness characteristics important for veterinary workplace clinical training: a novel application of pairwise comparisons and the Elo algorithm
Source: Front Med (Lausanne). 2023 Apr 21;10:1128058. doi: 10.3389/fmed.2023.1128058 (PMC10160665; doi:10.3389/fmed.2023.1128058)
Supplement: Supplementary file 1 [file Data_Sheet_1.docx]

Supplementary Material 1

Rating and ranking preparedness characteristics important for veterinary workplace clinical training: a novel application of pairwise comparisons and the Elo algorithm

**Jennifer Routh, Sharmini Julita Paramasivam, Peter Cockcroft, Sarah Wood, John Remnant, Cornélie Westermann, Alison Reid, Patricia Pawson, Sheena Warman, Vishna Devi Nadarajah, Kamalan Jeevaratnam^*^**

*** Correspondence:** Kamalan Jeevaratnam drkamalanjeeva@gmail.com

# Joint display

# Supplementary material 1 is a joint display used to map the qualitative dimensions of the group interview research^1^ (participant quotes) to the survey items. It provides evidence for how the survey items were systematically developed.

^1^Routh, J., Paramasivam, S.J., Cockcroft, P. *et al.* Stakeholder perspectives on veterinary student preparedness for workplace clinical training – a qualitative study. *BMC Vet Res***18**, 340 (2022). https://doi.org/10.1186/s12917-022-03439-6

| Quote | Preparedness characteristic (survey item) |
| --- | --- |
| *“That is something that I think is quite interesting, whether we should talk to them a bit more about what they might see in practice…everyone has got their own sort of challenges, and that’s quite normal.”*  *“To have an awareness of veterinary practice, the realities of it”* | Students' awareness of the challenges and realities of practice for veterinary practitioners |
| *“you need to know the professional norms and cultural norms”* | Students' awareness of the complex professional and cultural norms of the veterinary workplace |
| “*Our mentors in practice… their primary role is essentially being a vet in that practice …Their primary role is not to teach our students… so I think it’s even more important that the students recognise that”*  *“Understanding some of the tensions around the teacher who is wanting to focus on the task but also focus on the student needs”* | Students' awareness that their supervisor holds two roles (veterinary surgeon and teacher), and these sometimes compete with each other |
| *“A bit of commercial awareness would be nice actually….I think they do need some idea of how businesses run as well as how much things cost to owners, and how much things cost to buy.”* | Students' awareness of the commercial aspects of veterinary practice |
| *“I certainly have had conversations with students where they’ve talked about the challenge of rotations where they move… from practice to practice really quickly, and how difficult they find that because they just find their feet and then they move on”* | Students' awareness of the challenges associated with moving workplace clinical training location frequently |
| *“I think an awareness of workplace culture is actually a really important thing for students to have because if they get that different workplaces work in different ways, and again it’s just reflective practice isn’t it, it’s just them going in and thinking “oh my goodness, this is so different to where I was last week, I wonder why it’s different? Oh, it’s different for these reasons.””* | Students' awareness that there is variation between different workplaces |
| *“I think something that will help the students is to have sort of a flexibility and adaptability to different situations if they’re going to be part of a team.”* | Flexibility and adaptability |
| *“just observing and taking it all in really, in terms of what’s happening and…what’s going on.”*  *“it’s that engagement and that alertness … having that attention and that focus there I think is quite important”* | Observing what's going on in the workplace; an attentiveness |
| *“getting involved with all the members of the team that they’re working with, be that vets, nurses, receptionists, you know, I guess trying to put themselves forward to be involved or carry out tasks or help in whatever way they can”*  *“pitching in as a team member… offering to help out anybody in the team.”* | Teamwork, students putting themselves forward or offering to help |
| *“I think that social awareness helps them then when something is directed at them to go: “actually, I’ve seen that vet be flat out all day…””*  *“Perceptive, that’s an interesting quality isn’t it, that we probably do want from our vet students.”* | Social awareness, socially perceptive |
| “*I have increasingly started to tell my tutees that, to think on diplomacy rather than competitiveness”* | Diplomacy |
| *“I think it’s definitely big jump, because I think the biggest thing is the change in your role, and the change in the responsibility that you have, it’s quite different, and that’s something you can’t really learn.”* | Students' awareness of their own roles and responsibilities |
| *“The thing that crossed my mind, and again it sort of goes through everything and probably goes back to the code of professional conduct, is honesty as well”*  *“I think integrity and kind of dependability are big ones”* | Honesty, integrity, dependability |
| *“They have to develop a little bit of maturity around those feedback seeking behaviours.”*  *“It’s a real sign of maturity isn’t it, how they treat the people who they consider to be lower down the pecking order.”* | Maturity |
| *“I think that attention to detail and actually caring about their patients needs… I think is really, really important.”* | Attention to detail |
| *“you’ve got to stay, to a degree, calm”*  *“They should have a framework there that means they can have a go without panicking”* | Calmness, level-headedness |
| *“Be on time or five minutes early”*  *“Time is a big one really isn’t it and using time efficiently”*  *“That’s my biggest bug bear actually. I can’t believe how slow – this is one of the most – in fact, this probably the most important attribute but - speed up!”* | Timeliness |
| “*I think they need to have some personal leadership over taking breaks and having lunch”* | Personal leadership over breaks and work patterns |
| *“You have to have this kind of extreme ownership of things as well. Just saying “Right, I’m going to sort it. I’m going to make sure that it’s sorted”…They cannot just be – it’s not a 9:00am to 5:00pm job with no responsibilities.”*  *“I want them to work hard throughout the day, not procrastinate, twiddle their thumbs, not finish things, not call somebody”* | Commitment to completion of tasks |
| *“They are expected to be well presented”*  *“I think with referral they very much expect you to be in your scrubs, everything quite clean cut… whereas I think – because I was on farm last – they were quite happy for you to rock up, as long as everything was clean biosecurity wise, they really weren’t all that worried about your appearance, if your waterproofs were a bit wrinkly, they really didn’t care.”* | Well-presented, wearing appropriate clothing |
| *“Have the right kit, have the pen that’s useful for someone, have a bit of paper, have a stethoscope, thermometer, have the kit that you would be expected to have”*  *“I think a requirement of any particular equipment, they need to find that out.”* | Bringing the correct equipment |
| *“Familiarity with the environment…this is where the catheters are, like you said, this is where stuff is. This is where you talk them for a walk…”* | Familiarity with the environment, e.g. where things are, how the computer system works |
| *“It would make life easier for yourself by being able to get from A to B.”*  *“I don’t drive in the UK so.. one thing I definitely had to take into consideration when looking at my IMR [WCT] rotations…was can I get to the practice by public transport, is that even possible or is it in the middle of nowhere?”* | Able to get to and from workplace clinical training independently |
| *“students are expected to abide by the code of conduct and what the RCVS says”* | Students' awareness of the content of their licensing body's code of conduct (e.g. RCVS Code of Professional Conduct, AVMA Principles of Veterinary Medical Ethics) |
| *“Practices do let you have a phone in your pocket these days…as long as you’re not on Facebook all day.”* | Appropriate use of mobile phones and the internet |
| *“there’s a certain experiential learning process, it’s not something that you just learn from a textbook or anything else, so you do need those experiences”*  *“They think that they are going to get it just by seeing the case and I think the other thing that I find frustrating and sometimes alarming is the lack of active following up of cases.”*  *“it’s that kind of awareness of how you learn”* | Students' awareness of how they learn during workplace clinical training; an active experiential process |
| *“The caseload will be what the caseload is, and their experience will be what their experience is”*  *“I think they need to recognise that as soon as you throw someone into a clinical setting, we’re not managing that setting, we’re not controlling the caseload.”* | Students' awareness of the variation in the caseload that they experience |
| *“I think it is really, really important that students are told about the importance of a team and that they can learn veterinary skills from every single member of the team”* | Students' awareness of the value of the entire veterinary team and how students can learn from all of them |
| *“an awareness of gaps in knowledge and in a positive way, not in a “I can’t do that” and then giving up, in a “I don’t know how to do that therefore I’m going to do something about it”, that’s a very good student, that can see gaps and fills them.”* | Identifying knowledge gaps and saying "I don't know that" |
| *“maybe you haven’t come up with all the answers, go and do some self-learning on it, be prepared to do that, and be prepared to say “Look, can I go and do a bit of research on that?””* | Filling knowledge gaps, self-directed learning |
| *“Either knowing that they can do it, or knowing to ask for help, perhaps? Confidence to have a go or asking for some direction if they haven’t done it.”* | Asking for help |
| *“Students that go into their rotations enthusiastic get so much more out of it”*  *“I do see sometimes with some clinical contexts that the student who hasn’t had any clinical experience and is really lacking in clinical skills, sometimes they’re not trusted by their clinicians to do stuff and they get a little bit shoved to the side.”*  *“students with us definitely get more out of it if they are, not pushy, but confident enough to say “Can I go and do that?” or asking what’s on, rather than just sitting quietly and waiting for someone to say “Can I come out with you?””* | Students' awareness that being proactive, enthusiastic, demonstrating competence, and confidence can bring them opportunities in the workplace |
| *“a real willingness to actively learn and to seek out learning opportunities”*  *“being proactive, so if you’ve got access to the diary you could look forward in the week and see what cases are coming in and kind of going to the vets that are assigned to those cases and actively asking to be involved”* | Proactive in seeking personal learning opportunities |
| *“ask lots of good questions”* | Asking appropriate questions |
| *“now it’s not just working together to produce some work on a clinical problem – it’s how you’re going to conduct yourself as a group within the practice.”*  *“I think that sort of group dynamic and group learning, that real collaborative learning, can be so powerful as well too.”*  *“I think the perfect rotation group has a mix of students with different skills and they teach each other, and very much support each other”* | Working and learning with other students effectively |
| *“We expect them to have read all of this preparatory information that they’re going to need”*  *“we give a wealth of material, which is maybe something that needs addressing from our end, but if they haven’t read the instructions to know how the rotation starts, I just feel like it doesn’t make a good impression.”* | Reading the preparation material provided |
| *“…and making sure they are organised at five o’clock the previous evening. Because it drives me mad; we get ones that’ll text me at seven o’clock at night and say: “Where should I be in the morning?””*  *“I think self-discipline is one that I find is quite important on IMR”* | Self-discipline and organisation |
| *“some of the students and their learning objectives, some of them are really impractical things that they’re putting on there”*  *“they come along having identified some learning objectives that they want to achieve…so I do usually try, on day one, to take five minutes to talk about those and whether they’re achievable, or whether we need to alter those a little bit.”* | Setting reasonable personal learning objectives |
| *“we can set some information out… making sure there’s core learning outcomes... They need to expect to know what may or may not happen on that rotation.”* | Students' awareness of their expected learning outcomes (set by veterinary school or licensing body) |
| *“We’re not expecting you to do this perfectly”*  *“Understanding that… you’re not going to get everything right every time and people – they will need to make mistakes to learn”*  *“You will learn way more from that failure than any of your successes”* | Students' awareness that perfection is not expected; failure or mistakes are likely, and they are part of the learning process |
| *“I think resilience is the best attribute that any student can have coming in. You can mould the rest, as long as there is enough to cope with your kind of guidance, I suppose so I think for me resilience is so important.”*  *“I guess what I mean is just able to cope with fairly low-level stresses is what I mean by resilience.”* | Resilience in the face of failure, low-level stress and the pressure of the workplace |
| *“the best feedback is that that you get on the hoof… students that proactively develop their own feedback seeking skills will benefit from that a lot more”* | Seeks feedback |
| *“being receptive to feedback without getting angsty and defensive about it”* | Receptivity to feedback, including critical or constructive feedback |
| “*RES3:…everybody complains we don’t give enough feedback apparently. We give a lot of feedback, but very little feedback is received by a student. So, they don’t see feedback.*  *INT: So, a well-prepared student would know feedback when they saw it?*  *RES3: Oh God, yeah. I would 100% agree with that.”* | Understanding what both formal and informal feedback looks like in the workplace |
| *“I think that’s a key thing that I think would be really great… it would be good if they were more reflective when they get into their placement year.”*  *“I think that reflection is kind of being toyed with and we’re trying to get to the stage where they can reflect well on their actions, but I think there is still a way to go before they integrate it into their everyday practice”* | Engaging in meaningful reflection |
| *“what I would want from a student to be able to do is to show that level of independent learning and that level of self-awareness”*  *“I think unprepared students don’t, they don’t tend to also have this self-awareness… you get some students at the end of week one that, their first week on IMR, are so in a nervous/anxious mess because they’ve just completely overestimated what they thought they were capable of”* | Self-awareness of limitations, strengths and weaknesses |
| *“I think it’s really important for self-confidence. I see a lot of students who, I think it’s only natural because we’re British, like “Oh I couldn’t possibly” … and it’s like “Just give it a go, I think you probably can” and then they do it”* | Appropriate level of self-confidence |
| *“university is tough but getting into the clinics is tougher and I think at that point it could be a make or break time for someone with mental issues, so being able to recognise that in themselves, and being aware of that, and also kind of being aware of how to seek help, and how to self-care I think is important.”*  *“What is quite interesting I have found is students who are on IMR and have seen vets struggling with mental health issues in practice and they’ve kind of fed back to me to say “Oh, my mentor has been like this and they seem to be really struggling” and I think that’s really interesting that you can now identify that the profession is really tough”* | Students' awareness of their own and others' mental wellbeing, and the importance of self-care |
| *“seeing why they’re learning for themselves rather than for a grade”*  *“I certainly think we have some students who come through who are like I never want to see another horse in my life and they don’t care, they just want to pass and I’m not going to try, and they take as few cases as possible and that can be really hard work”* | Motivated to learn for a career in veterinary medicine, not for a grade or as a tick box exercise |
| *“on my second placement I was encouraged to be an animal advocate”*  *“I think for me the most important attribute that trumps all others is having compassion for the patients, because I think if they actually care about their patient, like truly care about their welfare and their comfort, and like all of – just recognise that their patient is a valuable and sentient being then everything else will fall into place”* | Animal advocate |
| *“I think a keen and enthusiastic attitude is very important.”* | Enthusiasm |
| *“the most important thing is you have a…willingness to get in there and give it a go with the support that you need”* | Willing to try new practical skills with appropriate support |
| *“I think for me it’s a kind of willingness to sort of throw themselves into all aspects of clinical practice even, and perhaps particularly if it’s not an area that they feel as confident in or they are as interested in”* | Open to learning about species not of particular career interest |
| *“I find it frustrating when they don’t realise that the skills that they’re learning on a farm are actually really helpful for small animals or for equine… that you can transfer those skills”* | Students' awareness of the transferability of skills learned during workplace clinical training |
| *“I guess good communication skills, it’s a bit of a cliché but I guess that’s something really important… communication with the team.”* | Team communication skills |
| *[They would] listen to you.* | Listening to the clinical supervisor |
| *“Especially because you’re there for such a short amount of time, you don’t want to take things too far when you don’t really know the people. And I think they prefer it if you were a bit more, not reserved because you still want to show personality, but just be more respectful with the things that you’re talking about.”* | Polite, respectful |
| *“I suppose I think about the way their face communicates with me when I first meet them. So, it’s warmth, friendly, smiley.”*  *“I had the feedback from the clinician … they are going to make really good vets because they’re personable”* | Personable and friendly |
| *“in fact at the clinic we’re like “we don’t care if you can do the surgery, if you can explain to the owner” and actually it’s part of their surgery teaching, you don’t have to be good at surgery, you have to be good at communication”*  *“Well they need to be competent at taking histories and attentive listening skills, giving information clearly and coherently, with client friendly language, able to participate in shared decision making with clients.”* | Client communication skills - able to deliver and discuss information |
| *“being able to use a telephone. I know that sounds stupid but part of their communication tutorials at uni and stuff, if that could incorporate telephones as well because it’s a really widespread problem. Really widespread.”* | Telephone skills |
| *“the personal attribute I’d like to see is empathy”*  *“Do you know what I’m thinking is the consideration and the kindness and care towards clients”* | Empathy, compassion, kindness |
| *“I think taking – being able to have a go at taking consult is a good skill to have. I know obviously you will definitely improve as you go through them and it’s scary at the beginning but being able to take a history, like a general one, to at least then think of a kind of problem list, even if you’re not able to then straight away think of all the diagnostics and treatment plan, at least be able to do that initial discussion with the client to build a good rapport and get enough information that you need to, to then maybe discuss it with the vet after”* | Able to structure and lead a consultation including history taking |
| *“I think I would expect them to do sort of follow up questions with the history to try and dig a little bit deeper into what the client has said”* | Listening and reacting with appropriate follow up questions during history taking |
| *“I think an easier topic to deal with is written communication, so that’s clinical notes mostly, and actually I do expect them to know how to write clinical notes according to what the code of conduct suggests and cover all the details”* | Written communication skills |
| *“I think it’s about having the framework for the approach for a pretty standard simple common case but so well embedded that you almost don’t have to think about that anymore”* | Having a clinical reasoning framework for common problems |
| *“I think they should be able to start to make and record assessments about their patients, not just the facts about them, but actually what they think that means for the patients”*  *“being able to assimilate written data and glean the most important things from it”* | Able to assimilate and understand the importance of clinical information in the case |
| *“come up with independent thought processes”*  *“I definitely got told as a student “It’s really refreshing having a student that tells me exactly their thought processes the whole way through”.”*  *“if they can take that logical approach and apply that, we’ll probably be able to have a reasonable conversation about anything”* | Logical, independent thought processes and making sensible attempts to reason |
| *“what happens if the owner can’t afford it? or is too worried about the general anaesthetic? what are you going to do then? So, I already see some evidence that they’re trying to prepare the students for owners, but I think that’s probably the area where they’re least prepared, is how you deal with this immense variation of owners.”*  *“I think final year is all about getting their knowledge, and that kind of scaffolding of clinical, professional reasoning married together…I think professional reasoning… brings in things like ethics and costs and clients, relationships with clients, dog, you know, features of the patient that are not anything to do with…their clinical presentation”* | Taking into account non-medical, owner or contextual factors during clinical decision making |
| *“For me what the rotations brings that we don’t well in curricula yet is … reasoning in the face of multiple different things going on. Different problems.”* | Clinical reasoning skills when faced with multiple clinical problems |
| *“they need some common differentials for the common things that present”* | Knowledge of common differential diagnoses |
| *“I think being able to perform a systematic clinical exam…think about what you’re finding to add to the problem list or strike off the problem list”*  *“They do need problem solving abilities actually”*  *“I think it’s recognition of clinical signs and drawing up of a sensible diagnosis, or the differential diagnosis”* | Problem solving and forming problem and/or differential diagnoses lists |
| *“Irrespective of what the context is, that they would seek the evidence, or they would critically evaluate something, just double check the facts on something rather than just saying “Oh, is that what you said? Okay, fair enough, that’s what the situation is”.”* | Engaging with evidence based veterinary medicine (EBVM) |
| *“I think it would be good if some of our students were a little bit further down the line on that [clinical reasoning]… the ability to justify and rationalise decision making.”* | Capable of proposing justified and rational clinical decisions |
| *“we need to really help students kind of grasp the idea, that they will be dealing with uncertainty for the rest of their lives. You know, that is the mainstay of veterinary work, it is actually, often we are dealing with uncertainty.”* | Students' awareness of uncertainty and risk in clinical decision making |
| “*it’s about just being aware that different people have different ways of doing things.”*  *“having an appreciation that everybody can do something a bit differently”* | Students' awareness that there's more than one way of doing something |
| *“I think anatomy is really important.”* | Appropriate knowledge of anatomy |
| *“I think pharmacology and therapeutics and I think that’s also known from medicine to be a knowledge that they find quite a barrier.”* | Appropriate knowledge of pharmacology and therapeutics |
| *“you’re not going to be able to deal with a cardiac case unless you have an understanding of the physiology and pathophysiology of the heart.”* | Appropriate knowledge of physiology |
| *“…just a bit on husbandry…we have some clueless on different production systems and things. Again, it’s quite difficult to progress into the clinical picture if you are still struggling to get your head around the system, full stop.”* | Appropriate knowledge of animal husbandry and production systems |
| *“And the parasitology knowledge is atrocious. Absolutely atrocious.”* | Appropriate knowledge of parasitology |
| *“the general core vaccines… [if] I said, “What are we vaccinating against?” I’d probably expect someone at that stage to be able to at least name the cause."* | Appropriate knowledge of the core vaccines for the principal domesticated species |
| *“…their ability to kind of integrate their own knowledge and apply it to clinical cases…I think that is definitely one of the things I see as the biggest jump going into final year, their ability to not just sort of tell me everything know about a case but ability to pull lots of knowledge from lots of different places and apply it to the case in front of them”* | Integrating and applying knowledge to cases |
| *“I don’t expect them to have perfect clinical skills, so giving injections, intubation, everything else, I expect them to know how to do things.”* | Know how to perform practical skills (and not necessarily be able to perform them) |
| *“a lot of our vet students are not necessarily confident and competent at handling horses, yet from day one you can’t really kind of do any of the rotation unless you can handle your patient”* | Competence and confidence handling animals |
| *“One thing I probably expect the student to know is how to safely handle the species you’re working with that’s safe for both you and the client, and the animal.”* | Working safely |
| *“I think the majority of students I probably expect can put a catheter in… maybe able to take bloods, but I would never be annoyed or disappointed if they couldn’t do any of those things”*  *“What they do in the clinical skills centre in preparing for clinical skills is really important.”* | Basic clinical skills e.g. blood sampling, placing an intravenous catheter |
| *“…develop some good manual dexterity which, even if it’s not being able to spay a cat, they’ve got the basic ability to handle the instruments to approach that appropriately.”*  *“the skills that I’m doing, which is holding my instruments correctly, handling my materials correctly, manipulating the tissue correctly, all of those things are fine.”* | Surgical dexterity and tissue handling |
| *“There would be some tasks, maybe taking an ear swab and looking at it under a microscope…some of the routine diagnostics that I would have hoped they would have done in clinical skills”* | Able to use diagnostic equipment e.g. use a microscope |
| *“One thing I did this year [in pre-clinical teaching] as an extension activity… was pick one of these drugs and go and look it up in NOAH. Just to see what the datasheet looks like. We should be doing a lot more of that so people just get used to not trying to remember everything, but just having a quick look in the formulary or NOAH.”*  *“I would expect them though to be competent at handling drugs and…calculating what they want to give”* | Able to use a formulary or product datasheets, and calculate drug doses |
| *“Being able to do a clinical exam”*  *“I think I would expect them to be able to do a basic physical exam”* | Clinical/physical examination skills |
| *“they do need the basic knowledge of what normal is”*  *“the basic TPRs for whatever species they’re looking at and normal parameters I think would just, we’d expect them to know.”* | Appropriate knowledge of what's normal on a clinical exam e.g. temperature, pulse and respiration rates |
