# Supplementary material for: Rating and ranking preparedness characteristics important for veterinary workplace clinical training: a novel application of pairwise comparisons and the Elo algorithm
Source: Front Med (Lausanne). 2023 Apr 21;10:1128058. doi: 10.3389/fmed.2023.1128058 (PMC10160665; doi:10.3389/fmed.2023.1128058)
Supplement: Supplementary file 2 [file Data_Sheet_2.docx]

Supplementary Material 2

Rating and ranking preparedness characteristics important for veterinary workplace clinical training: a novel application of pairwise comparisons and the Elo algorithm

**Jennifer Routh, Sharmini Julita Paramasivam, Peter Cockcroft, Sarah Wood, John Remnant, Cornélie Westermann, Alison Reid, Patricia Pawson, Sheena Warman, Vishna Devi Nadarajah, Kamalan Jeevaratnam^*^**

*** Correspondence:** Kamalan Jeevaratnam [drkamalanjeeva@gmail.com](mailto:drkamalanjeeva@gmail.com)

The Elo Rating System

An introduction to the Elo rating system including a step-by-step demonstration is available to watch at the link below. This video covers the content of sections 1-3 in this supplementary material.

<https://bit.ly/EloIntroandDemo>

# The football analogy

In the English football (soccer) leagues, teams play matches against each other and gain three points for a win or one point for a draw. At the end of the season, the team with the most points is deemed the best, and those with the least points are deemed the worst. The strength of the team is relative to the other teams in that league. In other words, the points a team in the Premier League has means nothing when compared to the points of a different team in a different league.

Imagine if, instead of always gaining three points for a win, teams gained points proportional to the unexpectedness of that win. So, if a team at the bottom of the league beat the top performing team at the time (a highly surprising result), they would gain many points, but if that better team beat the other (a highly predictable result), their points gain would be far less. Additionally, what if losers lost points? Again, this could be related to the expectation of the who would win and lose the match. So, if when two mid-table teams play each other, one team would win whilst the other would lose an equally moderate number of points. One can also think of this scenario as a “points exchange”; after a match the two teams exchange points, directly proportional to the unexpectedness of the outcome. In this imaginary scenario, a draw would not be an option.

This is how the Elo rating system works. However, instead of football teams the specific use-case described in this methods paper has preparedness characteristics (items) and instead of football matches, there are pairwise comparison questions in a survey.

# The underlying mathematics

At the beginning of data processing when the Elo algorithm is begun, all items have the same arbitrary score (zero). One pairwise comparison is processed at time i.e., sequentially rather than in parallel. For both items in that comparison, equation one calculates the probability of that item winning. This is called the ‘expected outcome’.

$$Expected outcome for item A=\frac{1}{\left( 1+ {10}^{{{((Existing rating}_{\left( item B \right)}-{Existing rating}_{\left( item A \right)})}/{400)}} \right)} (1)$$

$$Expected outcome for item B=\frac{1}{(1+ {10}^{{{((Existing rating}_{\left( item A \right)}-{Existing rating}_{\left( item B \right)})}/{400)}})} (1)$$

The expected outcomes of the two items in any pairwise comparison sum to one. The expected outcome is determined somewhat by the existing rating scores of the items in question. When the ratings of the items are the same, which most frequently occurs at the beginning of the data processing, the probability of either entity being chosen, or winning, is 0.5. Logically, when an item has a higher rating than its opponent, the expected outcome is greater than 0.5 and its magnitude is determined by the difference in the items’ ratings (i.e., how much ‘better’ the item is already judged to be).

Subsequently, equation two calculates the updated rating for each item. This is determined by the ‘expected outcome’ calculated in equation one, and whether the item ‘won’ or ‘lost’ the pairwise comparison.

$$Updated rating for item A= Old rating+K \times\left( S -expected outcome for A \right) \left( 2 \right)$$

$$Updated rating for item B= Old rating+K \times\left( S -expected outcome for B \right) \left( 2 \right)$$

$$S=0 if the item loses, 1 if it wins$$

$$k=100$$

The two-step process described above is completed sequentially for every pairwise comparison completed by all participants (Fig. 1). To be clear, ratings are not generated on a per participant basis, but the pairwise comparisons from all participants of interest are pooled together to generate ratings according to perspectives of that group.

Once all the pairwise comparisons have been processed, the Elo ratings should form a final stable ranking provided that 1) items are paired randomly, 2) there is sufficient variation in the importance of the items and 3) there is a reasonable degree of shared perspectives among participants in the group of study^1^.

Figure 1: The iterative process of generating characteristic/item ratings from pairwise comparison outcomes using the Elo rating system

# mElo ratings

One of the subtleties of the Elo rating system, is that the order in which pairwise comparisons are taken from the pool/‘bucket’ (Fig 1.), matters. To eliminate the effect of order, the entire process can be run many (X) times, with the order in which the pairwise comparisons are processed randomised each time. This will generate X Elo ratings for each item and this set of ratings will have a degree of variation, centered around a mean Elo (mElo) rating.

# Justification of parameters in the Elo rating system

## Equation constants

The parameter $\varepsilon=400$ (equation 1), which affects the spread of the ratings, is taken from the conventional wisdom of the rating system’s original use (generating rankings of chess players). In general, for every 400 rating points of advantage that A has over B, the probability of A beating B is expected to be 10 times greater than the probability of B beating A. However, this is irrelevant if the mElo ratings are min-max scaled (i.e., the spread is limited to between zero and one).

*k* (equation 2) is a constant representing the maximum point exchange. The exact value of *k* effects the volatility of system, i.e., how quickly the ratings change with each pairwise comparison. As *k* increases, winners gain more points and losers lose more points, and the impact that a surprising result has is greater. If *k* is too low, the ratings won’t change meaningfully as more pairwise comparison data is entered into the system. If *k* is too high, the ratings won’t stabilise sufficiently. However, given a sufficient number of pairwise comparisons, *k* has been demonstrated to have a limited effect on eventual item rankings^2, 3^. *k* is usually set between 16 and 200^3^, it has been set at 100 for the specific use-case described.

## 4.2 mElo randomisation number (X)

The mElo randomisation number (X) is the number of times that the Elo rating system is run. Each time the same pairwise comparison data is used, but the order in which the pairwise comparisons are entered into the system is randomised. It could be easier to think of this as X number of virtual sequences of the pairwise comparisons which are fed into the algorithm. This generates a set of X Elo ratings for each item. Each set will have a distribution of values due to the effect of sequence order, and the mean (mElo) rating is subsequently calculated. The value of X requires justification and optimisation.

Using a resampling with replacement method (bootstrapping), the 95% confidence intervals around mElo ratings were calculated with different values of X, using the pairwise comparison data from the first 412 completed ‘preparedness for veterinary workplace clinical training’ surveys. As X increased, the confidence intervals around mElo ratings decreased in width and the variation in width across items decreased, up to a point of plateau (Table 1). This point of plateau dictated the optimum mElo randomisation number (X=500). This is because increasing X beyond 500 randomisations would increase the compute time to run the Elo rating system without providing superior confidence in the values of mElo ratings.

| *Table 1: Mean and standard deviation mElo confidence interval width, across items, with different values of X (randomisation number)* | | |
| --- | --- | --- |
| **X** | **Mean mElo confidence interval width (across items)** | **mElo confidence interval standard deviation (across items)** |
| 1 | 404.52 | 17.36 |
| 10 | 153.30 | 8.49 |
| 100 | 97.45 | 7.59 |
| **500** | **90.75** | **7.82** |
| 1000 | 89.77 | 7.46 |
| 2000 | 89.63 | 8.14 |

## 4.3 Estimating the minimum number of questionnaire participants required

To generate trustworthy mElo ratings and rankings of items, their values should be relatively stable at the point at which all pairwise comparisons have been entered into the system. Therefore, the minimum number of pairwise comparisons (and participants) should be purposefully decided upon and justified.

The ‘EloChoice’ R package^4^ allows for the calculation of a *consistency index^1^*. The consistency index is, within a single run of the Elo rating system, the proportion of pairwise comparison outcomes that go in the direction of the expectation. In other words, it is the proportion of responses where the item with the higher Elo rating at the time is chosen, an unsurprising result. Given that the ratings are generated from the results of previous pairwise comparisons (Fig. 1) when there is a violation of the expected outcome, this is judged as inconsistency.

The consistency index is a value between 0 and 1, where 0 represents no agreement between pairwise comparisons and 1 represents complete agreement.

The index is calculated for a single run of the Elo rating system:

$$Non-weighted consistency index=1-\frac{\sum_{i=1}^{N} u_{i}}{N} (3)$$

$$Weighted consistency index=1-\sum_{i=1}^{N} \frac{u_{i}\times w_{i}}{\sum w} (4)$$

Where $u$ is a vector of length $N$ made up of 0s and 1s, where 1 indicates that the outcome of a pairwise comparison was *not* predicted by the existing Elo ratings, and 0 indicates that the outcome *did* match the expectation. $N$ is the total number of pairwise comparisons in that run of the Elo rating system for which a prediction was able to be made. Not all pairwise comparisons had an expected outcome either way. This occurs early in the run of the Elo rating system where the difference between the items’ rating scores is 0. $w$ is the absolute difference in Elo ratings between the two items in a pairwise comparison.

The package produces a non-weighted and weighted consistency index (equation 3 and 4, respectively). In the simpler non-weighted index, every pairwise comparison which is processed in the Elo rating system is simply assigned the binary score (0,1) depending on whether the outcome matches what the current ratings would predict the outcome to be. In the weighted consistency index, weight is given to the absolute Elo rating difference between the items being compared, so bigger ‘upsets’ (surprising results) contribute stronger to decreasing the index than smaller upsets.

The consistency index calculation is repeated for all the runs of the Elo rating system which contribute to the mElo ratings (n=500 in this use-case) and the mean weighted and non-weighted consistency indices are calculated.

Given that raters’ responses are pooled prior to running the Elo rating system (Fig. 1), it is important to acknowledge that inconsistency can be between- and within- raters. If there was only a single rater, then the consistency indices measure only intra-rater consistency, and if many raters performed only a single comparison each, the consistency indices measure only inter-rater consistency. In this use case the number of raters will depend on the response rate, but each perform fifty pairwise comparisons. The bias of the consistency indices towards measuring inter- or intra-rater consistency will depend on the ratio of raters to the number of times a rater makes a judgement on an item^1^.

The ‘raterprog’ function within the EloChoice package calculates the weighted consistency index with progressive rater inclusion. First, the weighted consistency index is calculated using *one* rater’s pairwise comparison outcome data. Then the weighted consistency index is calculated again, this time using the first *two* raters’ pairwise comparison outcome data. Then again, with the first *three* raters and so on until all raters in the group are included. This entire process is repeated ten times with the order in which the raters are included randomised each time. The median and quartiles of the weighted consistency indices of the ten repetitions are plotted (Fig 2. in blue), alongside the weighted consistency index progress using a single repetition with the original order of raters only (grey).

Fig 2. uses the pairwise comparison data from the first demographic group to be administered the survey (University of Surrey veterinary students, n=134). It demonstrates how the weighted consistency index increases rapidly and plateaus with increasing number of raters. This was used to determine the minimum number of participants required to generate trustworthy rankings of items. By twenty participants the weighted consistency index has plateaued; adding more raters would not significantly change the consistency of the pairwise comparison outcomes in the pooled set. This was set as the absolute minimum number of participants required per demographic group of interest. However, given that this threshold was generated from the data of a single demographic group, and other groups may have more varied responses, forty participants per group was aimed for.

Figure 2: A chart to demonstrate the weighted consistency index with increasing numbers of University of Surrey student raters included in the calculation.


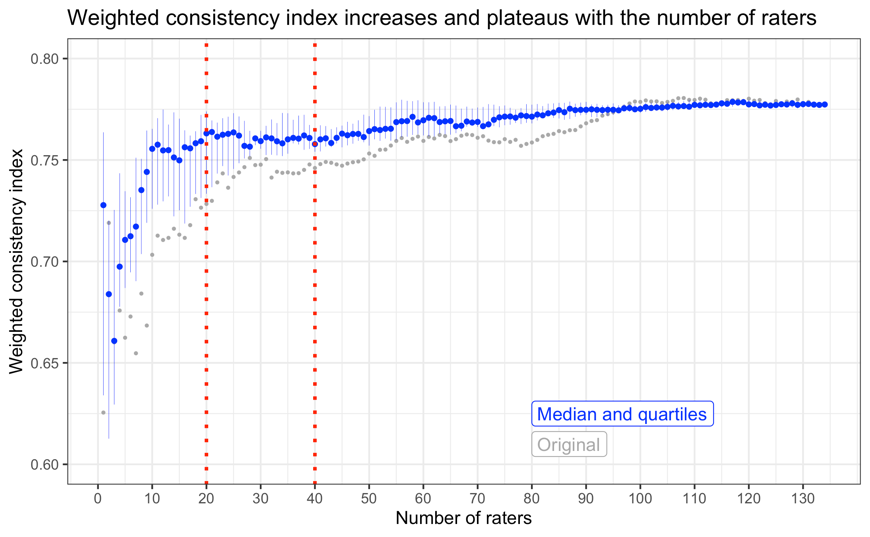


**References**

1. Clark AP, Howard KL, Woods AT, et al. Why rate when you could compare? Using the "EloChoice" package to assess pairwise comparisons of perceived physical strength. *PLoS One* 2018;13(1):e0190393-e.

2. Albers PCH, de Vries H. Elo-rating as a tool in the sequential estimation of dominance strengths. *Anim Behav* 2001;61(2):489-95.

3. Neumann C, Duboscq J, Dubuc C, et al. Assessing dominance hierarchies: validation and advantages of progressive evaluation with Elo-rating. *Anim Behav* 2011;82(4):911-21.

4. Neumann C. 2019 EloChoice: Preference Rating for Visual Stimuli Based on Elo Ratings. <https://cran.r-project.org/package=EloChoice>. Accessed: 31/05/2022.
